# Neuronal Correlates of Hyperalgesia and Somatic Signs of Heroin Withdrawal in Male and Female Mice

**DOI:** 10.1523/ENEURO.0106-22.2022

**Published:** 2022-07-06

**Authors:** Yocasta Alvarez-Bagnarol, Renata C. N. Marchette, Chase Francis, Marisela Morales, Leandro F. Vendruscolo

**Affiliations:** 1 Neuronal Networks Section, Integrative Neuroscience Research Branch, National Institute on Drug Abuse, Intramural Research Program, Baltimore, MD 21224; 2 Neurobiology of Addiction Section, Integrative Neuroscience Research Branch, National Institute on Drug Abuse, Intramural Research Program, Baltimore, MD 21224; 3 Department of Anatomy and Neurobiology, School of Medicine, University of Puerto Rico, Medical Sciences Campus, San Juan, Puerto Rico 00936-5067

**Keywords:** neuronal activity, opioids, pain, reinforcement, reward, stress

## Abstract

Opioid withdrawal involves the manifestation of motivational and somatic symptoms. However, the brain structures that are involved in the expression of different opioid withdrawal signs remain unclear. We induced opioid dependence by repeatedly injecting escalating heroin doses in male and female C57BL/6J mice. We assessed hyperalgesia during spontaneous heroin withdrawal and somatic signs of withdrawal that was precipitated by the preferential μ-opioid receptor antagonist naloxone. Heroin-treated mice exhibited significantly higher hyperalgesia and somatic signs than saline-treated mice. Following behavioral assessment, we measured regional changes in brain activity by automated the counting of c-Fos expression (a marker of cellular activity). Using Principal Component Analysis, we determined the association between behavior (hyperalgesia and somatic signs of withdrawal) and c-Fos expression in different brain regions. Hyperalgesia was associated with c-Fos expression in the lateral hypothalamus, central nucleus of the amygdala, ventral tegmental area, parabrachial nucleus, dorsal raphe (DR), and locus coeruleus (LC). Somatic withdrawal was associated with c-Fos expression in the paraventricular nucleus of the thalamus, lateral habenula, DR, and LC. Thus, hyperalgesia and somatic withdrawal signs were each associated with c-Fos expression in unique sets of brain areas. The expression of c-Fos in the DR and LC was associated with both hyperalgesia and somatic withdrawal. Understanding common neurobiological mechanisms of acute and protracted opioid withdrawal may help identify new targets for treating this salient aspect of opioid use disorder.

## Significance Statement

The public impact of the opioid crisis has prompted an effort to understand the neurobiological mechanisms of opioid use disorder (OUD). The need to avoid withdrawal symptoms is hypothesized to drive compulsive drug-taking and drug-seeking in OUD. Thus, understanding the mechanisms of acute and protracted opioid withdrawal may help identify new targets for treating this salient aspect of OUD. We reported brain structures that are associated with the expression of hyperalgesia and somatic signs of opioid withdrawal in male and female heroin-dependent mice. Hyperalgesia during spontaneous opioid withdrawal and somatic withdrawal resulted in c-Fos expression in autonomic and limbic brain regions. The expression of c-Fos in the dorsal raphe (DR) and locus coeruleus (LC) were associated with both hyperalgesia and somatic withdrawal.

## Introduction

Opioid use disorder (OUD) is a global burden with the highest estimated prevalence observed in the United States (GBD 2016 Disease and Injury Incidence and Prevalence Collaborators, 2017). The high prevalence of OUD has contributed to a national crisis. Nearly 92,000 individuals died from an opioid overdose in 2020 (National Institute on Drug Abuse, 2022). The public impact of the opioid crisis has prompted a national effort to understand the underlying neurobiological mechanisms of OUD. Opioid withdrawal is a physically and emotionally painful state that is hypothesized to drive continued drug taking and seeking and promote relapse in individuals with OUD. Opioid withdrawal signs include somatic and motivational signs, such as diarrhea, insomnia, fever, pain, anxiety, and depression (Koob, 2020, 2021). Somatic withdrawal varies in severity and duration, but it usually resolves within days or weeks, whereas motivational signs of withdrawal, such as heightened pain sensitivity (i.e., hyperalgesia), can persist for months or even years after the discontinuation of opioid use (Shurman et al., 2010; Carcoba et al., 2011).

Neuroimaging studies provide evidence of functional changes in brain regions during opioid withdrawal. Functional magnetic resonance imaging (fMRI) studies demonstrate that male OUD patients exhibit higher blood oxygen level-dependent (BOLD) signals in several brain regions, including the nucleus accumbens (NAc), caudate putamen, amygdala, hippocampus, prefrontal cortex (PFC), orbitofrontal cortex (OFC), medial frontal gyrus, thalamus, cingulate cortex, and subcallosal gyrus (Li et al., 2012; Murphy et al., 2018), during acute and short-term opioid abstinence. Greater subjective opioid craving scores correlated with greater activation of the NAc, caudate, putamen, and cingulate cortex (Murphy et al., 2018). A photon emission computerized tomography study identified lower blood flow in frontal and parietal cortices and higher blood flow in the thalamus in opioid-dependent male and female patients during naloxone-precipitated withdrawal (Krystal et al., 1995). In healthy males, naloxone administration increased neural activity in the pregenual cingulate, putamen, caudate, insula, hippocampus, and entorhinal cortex (Borras et al., 2004; Chu et al., 2015). The effects of naloxone on perceived pain intensity in response to a noxious thermal stimulus indicated greater activation of the insula, OFC, thalamus, and hippocampus (Borras et al., 2004). The assessment of objective and subjective symptoms of opioid withdrawal showed that naloxone had a minimal impact on withdrawal in healthy individuals, but changes in activity in the inferior orbital frontal gyri were associated with subjective withdrawal experience (Chu et al., 2015).

Preclinical studies identified associations between the activation of several brain regions and opioid withdrawal. In male morphine-dependent rats, naloxone administration increased c-*fos* mRNA expression in the hippocampus, lateral septal nucleus, periaqueductal gray (PAG), ventral tegmental area (VTA), locus coeruleus (LC), caudate putamen, NAc, bed nucleus of the stria terminalis (BNST), central nucleus of the amygdala (CeA), paraventricular nucleus of the hypothalamus (PVN), and lateral hypothalamus (LH; Delfs et al., 2000; Gracy et al., 2001; Frenois et al., 2002). Male morphine-dependent rats exhibited increases in glucose metabolism during naloxone-precipitated withdrawal in the CeA, the lateral habenula (LHb), the mamillary nucleus, the lateral septal nucleus, thalamic nuclei, and the interpeduncular nucleus (Wooten et al., 1982). An fMRI study in morphine-dependent rats revealed higher BOLD activity in the retrosplenial, piriform, insular, entorhinal, cingulate, visual, and auditory cortices and hippocampus (Lowe et al., 2002). Altogether, these clinical and preclinical studies provide evidence of the recruitment of several brain regions during opioid withdrawal, but they do not provide information about the potential role of these regions in specific behaviors. Moreover, results from spontaneous opioid withdrawal, which more closely mimics opioid withdrawal in humans (Azar et al., 2004; Papaleo and Contarino, 2006), are very limited.

Although sex differences in opioid dependence have been reported in humans and rodents (Zachry et al., 2019; Marchette et al., 2021; Pantazis et al., 2021), most studies were conducted in male subjects. Previous studies found that female opioid-dependent mice and rats exhibited similar levels of somatic signs of naloxone-precipitated heroin withdrawal compared with male opioid-dependent mice and rats (Cicero et al., 2002; Towers et al., 2019). A recent study reported an increase in c-Fos expression in the PAG, CeA, BNST, LHb, and paraventricular nucleus of the thalamus (PVT) following naloxone withdrawal-induced hyperalgesia in male and female oxycodone-dependent rats, but sex differences were not assessed (Simpson et al., 2020).

In the present study, we tested the hypothesis that hyperalgesia during spontaneous withdrawal and naloxone-precipitated somatic signs of withdrawal are associated with unique sets of brain region activity, but some of these regions are involved in both behavioral aspects of opioid withdrawal. We tested behavior and c-Fos expression in both male and female mice. We applied an unbiased approach to assess changes in neuronal activity throughout the brain that resulted from hyperalgesia during spontaneous opioid withdrawal and somatic signs that were precipitated by naloxone in male and female mice.

## Materials and Methods

### Animals

We used eight-week-old male and female C57BL/6J mice (The Jackson Laboratory). The mice were group-housed (three per cage) with the same sex and treatment condition (saline or heroin) in a temperature-controlled (23°C) vivarium with a 12/12 h light/dark cycle (lights on at 6:30 A.M.). We provided food and water *ad libitum* except during the experimental sessions. The study adhered to the National Institutes of Health *Guide for the Care and Use of Laboratory Animals* and was approved by the National Institute on Drug Abuse Animal Care and Use Committee.

### Drugs

We dissolved diamorphine hydrochloride (heroin; Research Triangle Institute), dispensed by the National Institute on Drug Abuse, Intramural Research Program Pharmacy, in sterile saline (0.9% sodium chloride; Hospira) at concentrations of 0.5–5 mg/ml. Heroin was administered subcutaneously in a volume of 10 ml/kg. We dissolved naloxone hydrochloride (DuPont Pharmaceuticals) in sterile saline at a concentration of 0.1 mg/ml. Naloxone was administered intraperitoneally in a volume of 10 ml/kg. We weighed the mice immediately before each injection with saline, heroin, or naloxone.

### Behavioral studies

#### Hyperalgesia during spontaneous heroin withdrawal

We assessed hyperalgesia by evaluating mechanical sensitivity using an electronic von Frey device (Ugo Basile). We habituated the mice to the testing room for at least 30 min. For acclimatization, we placed the mice in the testing apparatus for at least 1 h and started testing when exploratory behaviors ceased. The testing apparatus consisted of an elevated platform (92 cm in length × 28 cm in width × 71 cm in height) with a stainless-steel mesh floor (0.4 × 0.4 cm) and rectangular transparent compartments (10 × 10 × 13 cm) on top. For baseline measurements, we assessed mechanical sensitivity by applying a single unbending filament perpendicularly to the mid-plantar surface of either the left or right hind paw. The force at which the mouse retracted its paw in response to the stimulation [i.e., paw withdrawal threshold in gram-force (gf)] was recorded automatically by the von Frey device. We continued measuring the withdrawal threshold of the mice in adjacent boxes until we assessed withdrawal thresholds of each mouse at least once. We then repeated the process five times, alternating between the left and right hind paws until a total of six measurements for each mouse (three per paw) were collected. We calculated the average of the six measurements of paw withdrawal thresholds of each mouse and considered it as a measure of mechanical sensitivity.

**Figure 1.**
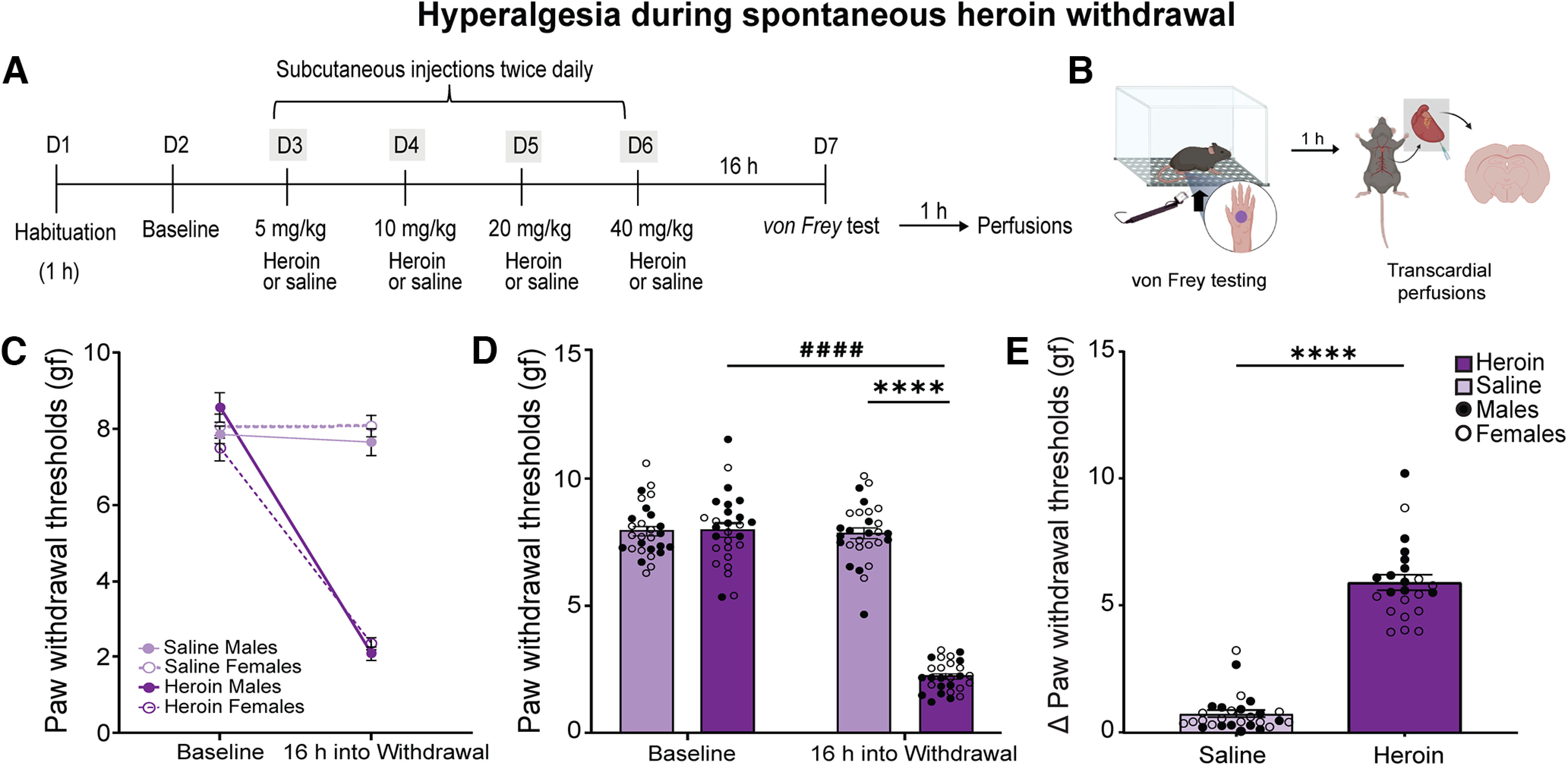
Repeated heroin injections caused mechanical hyperalgesia during withdrawal in male and female mice. ***A***, ***B***, Timeline and experimental procedures. Adult male and female mice were habituated for 1 h to the testing apparatus on day 1 (D1). We measured baseline paw withdrawal thresholds on D2. We gave mice twice daily subcutaneous injections of saline (10 ml/kg; nondependent) or increasing doses of heroin (5–40 mg/kg; dependent) from D3 to D5. Bodyweight loss during treatment is shown in Extended Data Figure 1-1. We tested paw withdrawal thresholds 16 h after the last heroin injection on D7 and perfused the mice 1 h later. ***C***, We did not detect significant differences between male and female mice in paw withdrawal thresholds at baseline and 16 h into withdrawal. We found significant effects of treatment (*p *<* *0.0001) and time (*p *<* *0.0001) and a significant treatment × time interaction (*p *<* *0.0001), indicating hyperalgesia in both sexes. The data are expressed as mean ± SEM *n *=* *13–14/group. ***D***, At 16 h into withdrawal, heroin-treated mice (males and females combined) had significant lower paw withdrawal thresholds compared with saline-treated mice (****p <* *0.0001) and compared with paw withdrawal thresholds during baseline (^####^*p *<* *0.0001). ***E***, Heroin-treated mice (males and females combined) exhibited significantly higher hyperalgesia during spontaneous opioid withdrawal than saline-treated mice (****p <* *0.0001). We calculated the Δ paw withdrawal threshold (paw withdrawal thresholds at baseline minus paw withdrawal thresholds at testing) to use as a single number for subsequent data analyses. The data are expressed as mean ± SEM *n *=* *27–28/group.

After obtaining baseline measurements, we randomly assigned the mice to the experimental groups. One group received injections of saline (10 ml/kg, s.c.), and the other groups received increasing doses of heroin (5, 10, 20, and 40 mg/kg, s.c.) twice daily (7 A.M. and 7 P.M.) for four consecutive days. Around 16 h after the last saline or heroin injection, we assessed paw withdrawal thresholds by von Frey testing as described above (Fig. 1*A*). To correlate behavior and c-Fos expression, we obtained a single numeric value that was representative of hyperalgesia by calculating the change (Δ) in paw withdrawal thresholds for each mouse. We obtained the Δ of paw withdrawal thresholds by subtracting paw withdrawal thresholds during withdrawal from paw withdrawal thresholds during baseline.

10.1523/ENEURO.0106-22.2022.f1-1 Extended Data Figure 1-1 Repeated heroin injections caused bodyweight loss in male and female mice. Percent of bodyweight change in mice that were used for the c-Fos expression experiment following the assessment of hyperalgesia during spontaneous heroin withdrawal. The Student’s *t* test showed that heroin-treated mice (male and female data combined) weighed significantly less than saline-treated mice (****p < 0.0001). The data are expressed as mean ± SEM. *N *=* *12/group. Download Figure 1-1, TIF file.

As an index of general opioid effects, we weighed the male and female mice daily across treatment. We calculated the total percentage of bodyweight loss by dividing the weight lost by the weight before the first injection multiplied by 100.

#### Naloxone-precipitated heroin withdrawal

**Figure 2.**
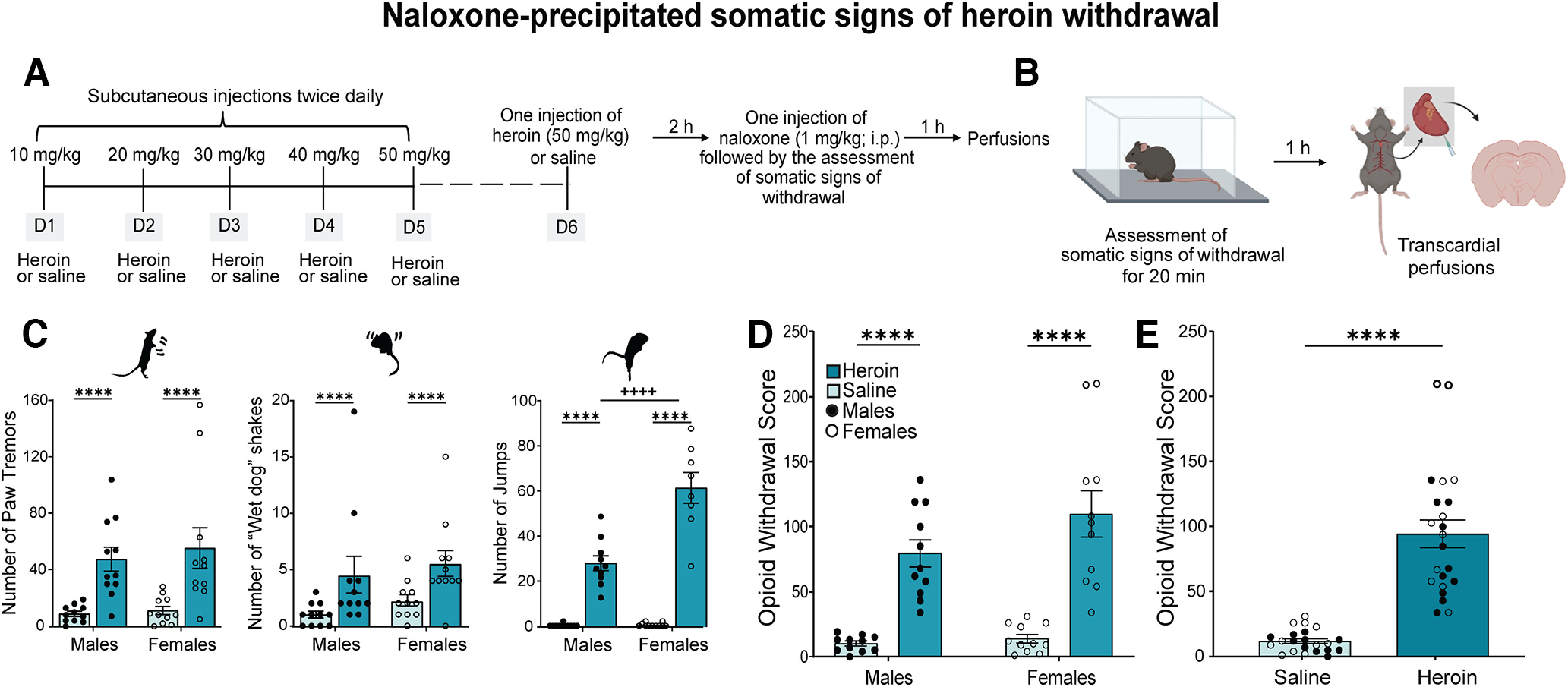
Repeated heroin injections produced naloxone-precipitated somatic signs of opioid withdrawal in male and female mice. ***A***, ***B***, Timeline and experimental procedures. We gave mice twice daily injections of saline (10 ml/kg, s.c.) or increasing doses of heroin (10–50 mg/kg, s.c.) from D1 to D5. On D6, we gave mice a single injection of saline or heroin (50 mg/kg, s.c.). Bodyweight loss during treatment is shown in Extended Data Figure 2-1. Two hours after the saline or heroin injection, we precipitated withdrawal with a single intraperitoneal injection of the preferential μ-opioid receptor antagonist naloxone (1 mg/kg) and recorded somatic signs of withdrawal for 20 min. We perfused mice 1 h after the end of behavior scoring (i.e., 80 min after the naloxone injection). ***C***, We did not detect significant differences between male and female mice in paw tremors or “wet-dog” shakes, but heroin-treated mice exhibited a significantly higher number of paw tremors (****p <* *0.0001) and “wet-dog” shakes (****p <* *0.0001) than saline-treated mice. Heroin-treated female mice exhibited a higher number of jumps compared with heroin-treated male mice (++++*p < *0.0001), and heroin-treated mice of both sexes exhibited a significantly higher number of jumps compared with saline-treated mice (****p < 0.0001). ***D***, We found an effect of treatment on opioid withdrawal scores (****p <* *0.0001), with no significant differences between male and female mice. We calculated the opioid withdrawal score (sum of the number of occurrences of individual somatic signs) to use as a single number for subsequent data analyses. The data are expressed as mean ± SEM *n *=* *11/group. ***E***, Heroin-dependent mice (male and female data combined) exhibited a higher opioid withdrawal score than saline-treated mice (****p <* *0.0001). The data are expressed as mean ± SEM *n *=* *22/group. gf, grams of force.

We injected mice with saline (10 ml/kg, s.c.) or increasing doses of heroin (10, 20, 30, 40, and 50 mg/kg, s.c.) twice daily (7 A.M. and 7 P.M.) for five consecutive days. On the day of the test (day 6 of the experimental timeline; Fig. 2*A*), we treated the mice with a single injection of saline or heroin (50 mg/kg). Two hours later, we injected the mice with the preferential μ-opioid receptor antagonist naloxone (1 mg/kg, i.p.) and immediately placed them individually in transparent cages to record somatic signs of naloxone-precipitated opioid withdrawal for 20 min. We counted the number of paw tremors (i.e., “clapping” front paws), jumps, and “wet-dog” shakes. We assigned one point per observation for each behavior. We also assigned one point per observation for the appearance of less frequent signs of withdrawal, such as abnormal posture, genital grooming, and diarrhea. To correlate behavior and c-Fos expression, we obtained a single numeric value that was representative of somatic withdrawal by summing the number of occurrences of these somatic signs.

10.1523/ENEURO.0106-22.2022.f2-1 Extended Data Figure 2-1 Repeated heroin injections caused bodyweight loss in male and female mice. Percent of bodyweight change in mice used for the c-Fos expression experiment following the assessment of naloxone-precipitated somatic signs of opioid withdrawal. The Student’s *t* test showed that heroin-treated mice (male and female data combined) weighed significantly less than saline mice (****p < 0.0001). The data are expressed as mean ± SEM. *N *=* *12/group. Download Figure 2-1, TIF file.

### Anatomical/histologic studies

#### Tissue preparation and c-Fos immunolabeling

We perfused mice 1 h after the end of behavioral testing to detect the expression of c-Fos protein (Figs. 1*B*, 2*B*). We anesthetized the mice with an injection of Euthasol (pentobarbital sodium; 100 mg/kg, i.p., 3 ml/kg) and transcardially perfused them with 4% paraformaldehyde (PFA) in 0.1 m phosphate buffer (PB). We kept the brains in 4% PFA for 2 h, then kept them in 18% sucrose solution overnight at 4°C. We collected free-floating coronal serial cryosections (16 μm thick) from the entire brain of each mouse with a cryostat (CM3050 S, Leica). To reduce c-Fos staining variability that can be caused by extraneous factors, we performed parallel immunohistological staining of a set of sections from mice of each behavioral cohort (i.e., male heroin, male saline, female heroin, and female saline).

We rinsed the cryosections with PB three times for 10 min each. We incubated the cryosections in 0.3% H_2_O_2_ for 15 min to quench endogenous peroxidase activity. Next, we rinsed and placed the sections for 1 h in blocking solution (4% bovine serum albumin and 0.3% Triton X-100 in PB). We incubated the sections overnight at 4°C with rabbit anti-phospho-c-Fos (1:2000; catalog #5348; Cell Signaling Technology) in blocking solution. After rinsing with PB three times for 10 min each, we incubated the sections for 1 h at room temperature with biotinylated goat anti-rabbit antibody (1:200; catalog #PK6101; Vector Laboratories) in blocking solution. We incubated the sections for 1 h at room temperature in avidin-biotinylated horseradish peroxidase (1:100, VECTASTAIN Elite ABC-HRP kit, Vector Laboratories). We developed the peroxidase reaction with 0.05% 3,30-diaminobenzidine (DAB) and 0.003% H_2_O_2_, rinsed the sections with PB three times for 10 min each, and mounted the sections on gelatin-coated slides. After drying overnight at room temperature, we dehydrated the sections through a series of graded alcohols, cleared the sections with Histoclear, and coverslipped the sections with Permount mounting medium (catalog #SP15-100; Fisher Scientific). Using an Olympus VS120 microscope (Olympus), we took brightfield images of whole sections that were magnified with a 20× objective.

#### c-Fos segmentation and atlas registration

For the automatic cell detection and counting of labeled c-Fos protein, we wrote two macros (available as Extended Data) using Image-Pro 10.0 software (Media Cybernetics). The automated macros quantified stained nuclei by extracting RGB values of the image and applying iterative morphologic segmentation. For the optimal detection of DAB staining, the macro preprocessed the images by performing spatial calibration and color extraction to view images as an eight-bit gray scale image. This allowed us to convert the original image to other color spaces to eliminate the correlation between RGB values and thus associate each image pixel to a different stain based on thresholds. We scaled the images to their representative plate in the mouse brain atlas (Konsman, 2003) to select and manually draw contours of regions of interest. We focused our analysis on subcortical regions that have been shown to be involved in withdrawal and negative affect (Maldonado et al., 1996; Welsch et al., 2020). To reduce variability between samples, we used a constant number of sections (4–10 per mouse, depending on the brain region) for each region of interest. We used another macro to automatically define thresholds to identify c-Fos-positive nuclei by defining the range of intensity values that distinguished them from background. To properly segment positive nuclei, the macro detected different morphologic ranges of areas based on pixel values and applied a watershed auto-split procedure of selected objects to separate clusters of positive nuclei. Finally, the macro exported the total number of positive nuclei per region of interest to an Excel spreadsheet.

### Statistical analysis

The number of mice (*n*) that were used in each group or condition is described in each figure legend. We analyzed paw withdrawal thresholds using three-way repeated-measures ANOVA, with sex and treatment (repeated heroin or saline) as between-subjects factors and time (test day) as the within-subjects factor. Because we found no significant group or time × sex interaction, we combined the male and female data and analyzed paw withdrawal thresholds using two-way ANOVA, with time and treatment as factors. We analyzed the Δ of paw withdrawal thresholds using Student’s *t* test. We analyzed somatic signs of withdrawal using two-way ANOVA, with sex and treatment as between-subjects factors. For opioid withdrawal scores, because we did not observe a significant sex × treatment interaction, we combined male and female data to present the main effect of treatment. We analyzed c-Fos expression after the assessment of hyperalgesia or somatic signs of withdrawal using two-way ANOVA, with sex and treatment as between-subjects factors. We present the combined data in the main manuscript and separate sex data in the Extended Data.

We conducted Principal component analysis (PCA), which standardizes and transforms data to comparable scales, with an orthogonal normalized Varimax to search for associations between c-Fos expression and the behavioral results. We retained factors with eigenvalues >1 and considered factor loadings ≥0.7 to represent variance that was explained by the factors. We used Pearson’s correlation test to analyze c-Fos expression among brain regions. We analyzed the data using Prism 9.2 software (GraphPad) or Statistica software (Statsoft).

## Results

### Hyperalgesia during spontaneous withdrawal after repeated heroin injections

To model opioid dependence and produce hyperalgesia during spontaneous heroin withdrawal in male and female mice, we used a 4-d schedule of repeated subcutaneous administration of escalating heroin doses (5–40 mg/kg). We measured paw withdrawal thresholds 1 d before initiating the heroin injections (baseline) and after repeated heroin injections at 16 h into withdrawal (Fig. 1*A*). The three-way ANOVA (sex × treatment × time) showed significant effects of treatment (*F*_(1,52)_ = 149.9, *p < *0.0001) and time (*F*_(1,52)_ = 312.6, *p < *0.0001), a significant treatment × time interaction (*F*_(1,52)_ = 288.2, *p < *0.0001), no effect of sex (*F*_(1,52)_ = 0.04,084, *p = *0.8406), no sex × treatment interaction (*F*_(1,52)_ = 2.516, *p = *0.1188), and no sex × treatment × time interaction (*F*_(1,52)_ = 2.610, *p = *0.1122; Fig. 1*C*). Because we did not observe significant interactions with the sex factor, we combined the male and female data. The two-way ANOVA (treatment × test day) showed significant effects of treatment (*F*_(1,53)_ = 215.5, *p < *0.0001) and test day (*F*_(1,53)_ = 268.9, *p < *0.0001) on paw withdrawal thresholds and a significant treatment × test day interaction (*F*_(1,53)_ = 252.5, *p < *0.0001; Fig. 1*D*). *Post hoc* comparisons indicated that heroin-treated mice had significantly lower paw withdrawal thresholds compared with saline-treated mice (*p < *0.0001) and compared with paw withdrawal thresholds during baseline (*p < *0.0001). Student’s *t* test of the Δ of paw withdrawal thresholds indicated that heroin-treated mice exhibited significantly higher mechanical sensitivity compared with saline-treated mice (*t*_(53)_ = 16.78, *p < *0.0001; Fig. 1*E*).

We measured bodyweight in mice that were subsequently used to detect c-Fos expression after the assessment of hyperalgesia. Student’s *t* test showed that heroin-treated mice weighed significantly less than saline-treated mice (*t*_(22)_ = 10.13, *p < *0.0001; Extended Data Fig. 1-1).

### Naloxone-precipitated somatic signs of withdrawal after repeated heroin injections

To test somatic signs of heroin withdrawal, we used a 5-d schedule of intermittent injections of escalating heroin doses (10–50 mg/kg, s.c.) and precipitated withdrawal by a single intraperitoneal injection of naloxone (1 mg/kg; Fig. 2*A*) 2 h after a heroin injection. Somatic signs of heroin withdrawal were assessed by counting the number of jumps, paw tremors (“clapping” of forepaws), shakes (“wet-dog” shakes), and other miscellaneous behaviors. The two-way ANOVA showed a significant treatment effect on paw tremors (*F*_(1,40)_ = 23.58, *p < *0.0001; heroin > saline), regardless of sex (sex effect: *F*_(1,40)_ = 0.3612, *p *=* *0.5512; sex × treatment interaction: *F*_(1,40)_ = 0.1053, *p = *0.7473; Fig. 2*C*). The two-way ANOVA also showed a significant treatment effect on wet-dog shakes (*F*_(1,40)_ = 10.75, *p = *0.0022; heroin > saline), regardless of sex (sex effect: *F*_(1,40)_ = 1.195, *p *=* *0.2809; sex × treatment interaction: *F*_(1,40)_ = 0.01,720, *p = *0.8963). The ANOVA showed significant effects of treatment (*F*_(1,40)_ = 196.6, *p < *0.0001; heroin > saline) and sex (*F*_(1,40)_ = 27.97, *p < *0.0001) on the number of jumps and a significant sex × treatment interaction (*F*_(1,40)_ = 27.07, *p < *0.0001). *Post hoc* comparisons indicated that female heroin-treated mice exhibited a higher number of jumps compared with male heroin-treated mice (*p < *0.0001).

The two-way ANOVA revealed that heroin-treated mice had a significantly higher opioid withdrawal score compared with saline-treated mice (treatment effect: *F*_(1,40)_ = 62.10, *p < *0.0001), regardless of sex (sex effect: *F*_(1,40)_ = 2.685, *p *=* *0.1092; sex × treatment interaction: *F*_(1,40)_ = 1.602, *p *=* *0.2129; Fig. 2*D*). Because we did not observe a significant sex × treatment interaction, we combined male and female data to present the main effect of treatment (Fig. 2*E*). These results suggest that naloxone precipitated somatic withdrawal in heroin-treated mice independently of sex.

We measured bodyweight in mice that were subsequently used to detect c-Fos expression after the assessment of somatic signs of withdrawal. Student’s *t* test showed that heroin-treated mice weighed significantly less than saline-treated mice (*t*_(22)_ = 8.945, *p < *0.0001; Extended Data Fig. 2-1).

### Increase in cellular c-Fos expression in heroin-dependent mice following the assessment of hyperalgesia during spontaneous withdrawal

**Figure 3.**
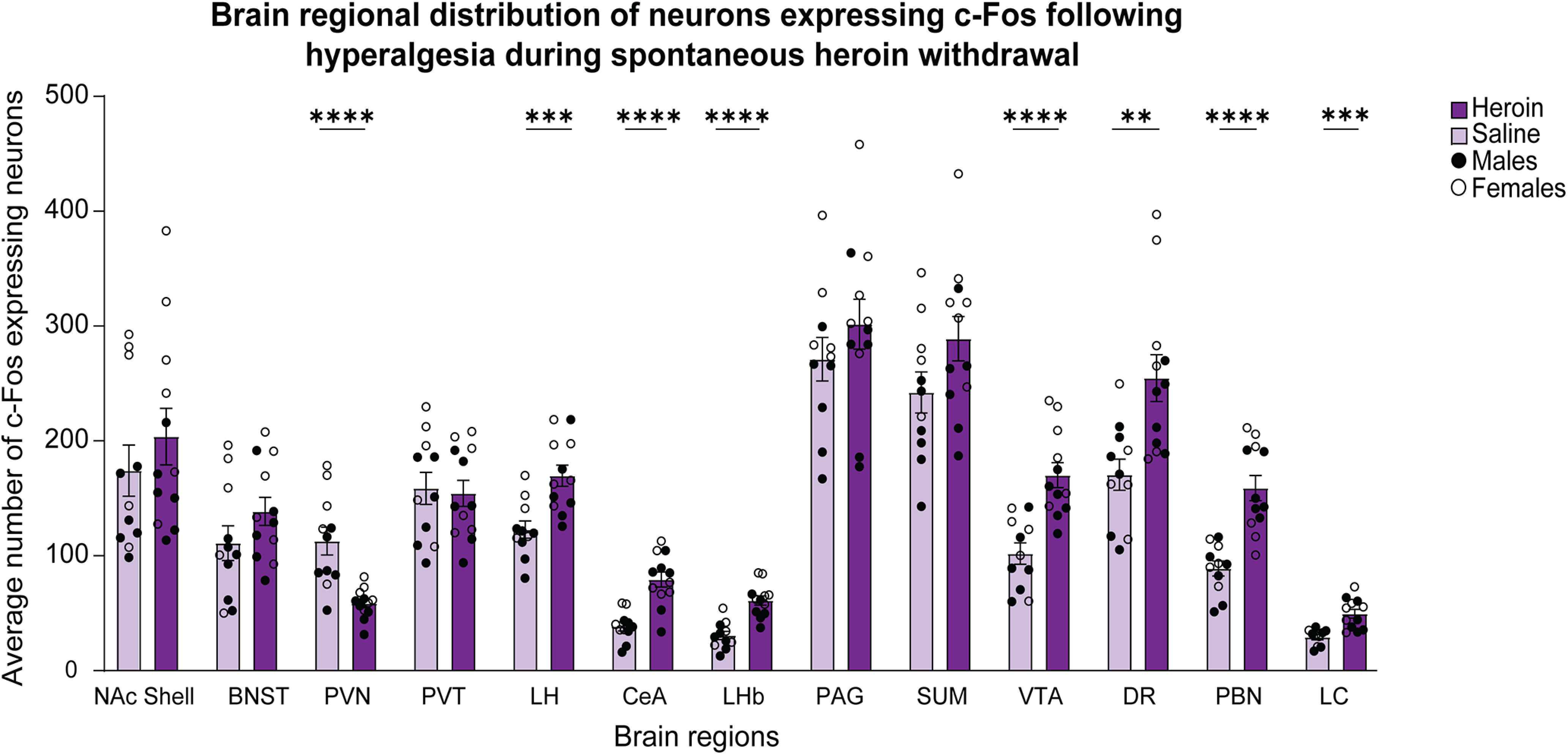
Regional distribution of c-Fos-expressing neurons following the assessment of hyperalgesia during spontaneous heroin withdrawal. Significant effects of treatment on c-Fos expression were found in the PVN (****p <* *0.0001), LH (***p <* *0.001), CeA (****p <* *0.0001), LHb (****p <* *0.0001), VTA (****p <* *0.0001), DR (**p <* *0.01), PBN (****p <* *0.0001), and LC (***p <* *0.001). The data are expressed as mean ± SEM *n *=* *11–12/group (male and female data combined). A comparison of c-Fos expression levels between heroin-treated and saline-treated male and female mice is shown in Extended Data Figure 3-1.

To investigate brain region-specific activity that is associated with hyperalgesia during spontaneous withdrawal, we quantified c-Fos expression in brains that were collected 17 h after the last heroin injection (1 h after von Frey testing; Fig. 1*A*,*B*). We compared the regional average of c-Fos expression between heroin-treated and saline-treated male and female mice (Extended Data Fig. 3-1). Statistical values are shown in Table 1. We found an increase in c-Fos expression in female mice compared with male mice in the NAc shell, PVN, LH, LHb, PAG, SUM, and VTA. The two-way ANOVAs revealed no sex × treatment interactions for any of the analyzed brain regions. We found effects of treatment (heroin > saline) on c-Fos expression in the LH, CeA, LHb, VTA, dorsal raphe (DR), parabrachial nucleus (PBN), and LC (Fig. 3). These results indicated that spontaneous heroin withdrawal significantly increased c-Fos expression in distinct brain regions in a sex-independent manner.

**Table 1:** Two-way ANOVA results of Fos quantification in analyzed brain regions following hyperalgesia during spontaneous withdrawal

Brain region	Treatment effect	Sex effect	Sex × treatment interaction
NAc shell	*F*_(1,19)_ = 0.8349, *p = *0.3723	*F*_(1,19)_ = 10.41, *p* = 0.0044; F > M	*F*_(1,19)_ = 0.05892, *p* = 0.8108
BNST	*F*_(1,19)_ = 1.926, *p = *0.1813	*F*_(1,19)_ = 4.275, *p* = 0.0526	*F*_(1,19)_ = 0.4395, *p* = 0.5153
PVN	*F*_(1,19)_ = 30.16, *p < *0.0001	*F*_(1,19)_ = 8.964, *p* = 0.0075; F > M	*F*_(1,19)_ = 0.2445, *p* = 0.1344
PVT	*F*_(1,19)_ = 0.1164, *p = *0.7367	*F*_(1,19)_ = 2.544, *p* = 0.1272	*F*_(1,19)_ = 0.2627, *p* = 0.6142
LH	*F*_(1,19)_ = 16.93, *p < *0.001	*F*_(1,19)_ = 5.546, *p* = 0.0294; F > M	*F*_(1,19)_ = 0.1327, *p* = 0.7197
CeA	*F*_(1,19)_ = 26.37, *p < *0.0001	*F*_(1,19)_ = 1.876, *p* = 0.1867	*F*_(1,19)_ = 0.09373, *p* = 0.7628
LHb	*F*_(1,19)_ = 40.80, *p < *0.0001	*F*_(1,19)_ = 8.195, *p* = 0.0100; F > M	*F*_(1,19)_ = 0.9071, *p* = 0.3528
PAG	*F*_(1,19)_ = 1.136, *p = *0.2998	*F*_(1,19)_ = 8.589, *p* = 0.0086; F > M	*F*_(1,19)_ = 0.005, *p* = 0.9441
SUM	*F*_(1,19)_ = 4.195, *p = *0.0546	*F*_(1,19)_ = 14.44, *p* = 0.0012; F > M	*F*_(1,19)_ = 0.007174, *p* = 0.9334
VTA	*F*_(1,19)_ = 27.39, *p < *0.0001	*F*_(1,19)_ = 5.773, *p* = 0.0267; F > M	*F*_(1,19)_ = 0.1231, *p* = 0.2810
DR	*F*_(1,19)_ = 11.70, *p < *0.01	*F*_(1,19)_ = 1.803, *p* = 0.1952	*F*_(1,19)_ = 0.08641, *p* = 0.3642
PBN	*F*_(1,19)_ = 26.15, *p < *0.0001	*F*_(1,19)_ = 0.2822, *p* = 0.6014	*F*_(1,19)_ = 0.1759, *p* = 0.6796
LC	*F*_(1,19)_ = 17.91, *p < *0.001	*F*_(1,19)_ = 0.5460, *p* = 0.4695	*F*_(1,19)_ = 0.5909, *p* = 0.4520

Males (M), Females (F).

10.1523/ENEURO.0106-22.2022.f3-1 Extended Data Figure 3-1 C-Fos expression in neurons distributed in distinct brain regions in male and female mice following the assessment of hyperalgesia during spontaneous heroin withdrawal. Two-way ANOVAs showed no sex × treatment interactions for any of the analyzed brain regions. We found main treatment effect and main sex effect on the number c-Fos-expressing neurons in several brain regions: LH (treatment effect: *F*_(1,19)_ = 16.93, *p < *0.001; sex effect: *F*_(1,19)_ = 5.546, *p *=* *0.0294; sex × treatment interaction: *F*_(1,19)_ = 0.1327, *p *=* *0.7197), LHb (treatment effect: *F*_(1,19)_ = 40.80, *p < *0.0001; sex effect: *F*_(1,19)_ = 8.195, *p *=* *0.0100; sex × treatment interaction: *F*_(1,19)_ = 0.9071, *p *=* *0.3528), VTA (treatment effect: *F*_(1,19)_ = 27.39, *p < *0.0001; sex effect: *F*_(1,19)_ = 5.773, *p *=* *0.0267; sex × treatment interaction: *F*_(1,19)_ = 0.1231, *p *=* *0.2810), PVN (treatment effect: *F*_(1,19)_ = 30.16, *p < *0.0001; sex effect: *F*_(1,19)_ = 8.964, *p *=* *0.0075; sex × treatment interaction: *F*_(1,19)_ = 0.2445, *p *=* *0.1344), and SUM (treatment effect: *F*_(1,19)_ = 4.195, *p = *0.0546; sex effect: *F*_(1,19)_ = 14.44, *p *=* *0.0012; sex × treatment interaction: *F*_(1,19)_ = 0.007174, *p *=* *0.9334). We found significant treatment effect but no sex effect in the following brain regions: CeA (treatment effect: *F*_(1,19)_ = 26.37, *p < *0.0001; sex effect: *F*_(1,19)_ = 1.876, *p *=* *0.1867; sex × treatment interaction: *F*_(1,19)_ = 0.09373, *p *=* *0.7628), DR (treatment effect: *F*_(1,19)_ = 11.70, *p < *0.01; sex effect: *F*_(1,19)_ = 1.803, *p *=* *0.1952; sex × treatment interaction: *F*_(1,19)_ = 0.08641, *p *=* *0.3642), PBN (treatment effect: *F*_(1,19)_ = 26.15, *p < *0.0001; sex effect: *F*_(1,19)_ = 0.2822, *p *=* *0.6014; sex × treatment interaction: *F*_(1,19)_ = 0.1759, *p *=* *0.6796), and LC (treatment effect: *F*_(1,19)_ = 17.91, *p < *0.001; sex effect: *F*_(1,19)_ = 0.5460, *p *=* *0.4695; sex × treatment interaction: *F*_(1,19)_ = 0.5909, *p *=* *0.4520). We did not find significant treatment effects, but we found main sex effects on the number of c-Fos-expressing neurons in three brain regions: NAc shell (treatment effect: *F*_(1,19)_ = 0.8349, *p = *0.3723; sex effect: *F*_(1,19)_ = 10.41, *p *=* *0.0044; sex × treatment interaction: *F*_(1,19)_ = 0.05892, *p *=* *0.8108), BNST (treatment effect: *F*_(1,19)_ = 1.926, *p = *0.1813; sex effect: *F*_(1,19)_ = 4.275, *p *=* *0.0526; sex × treatment interaction: *F*_(1,19)_ = 0.4395, *p *=* *0.5153), and PAG (treatment effect: *F*_(1,19)_ = 1.136, *p = *0.2998; sex effect: *F*_(1,19)_ = 8.589, *p *=* *0.0086; sex × treatment interaction: *F*_(1,19)_ = 0.005, *p *=* *0.9441). We did not find a main treatment effect nor sex effect in the PVT (treatment effect: *F*_(1,19)_ = 0.1164, *p = *0.7367; sex effect: *F*_(1,19)_ = 2.544, *p *=* *0.1272; sex × treatment interaction: *F*_(1,19)_ = 0.2627, *p *=* *0.6142). Download Figure 3-1, TIF file.

We also found that the average number of c-Fos-expressing cells was significantly lower in the heroin-treated group in the PVN. We did not find significant treatment effects on c-Fos expression in the NAc shell, BNST, PVT, PAG, or supramammillary nucleus (SUM).

### Increase in cellular c-Fos expression in heroin-dependent mice following the assessment of naloxone-precipitated somatic signs of opioid withdrawal

**Figure 4.**
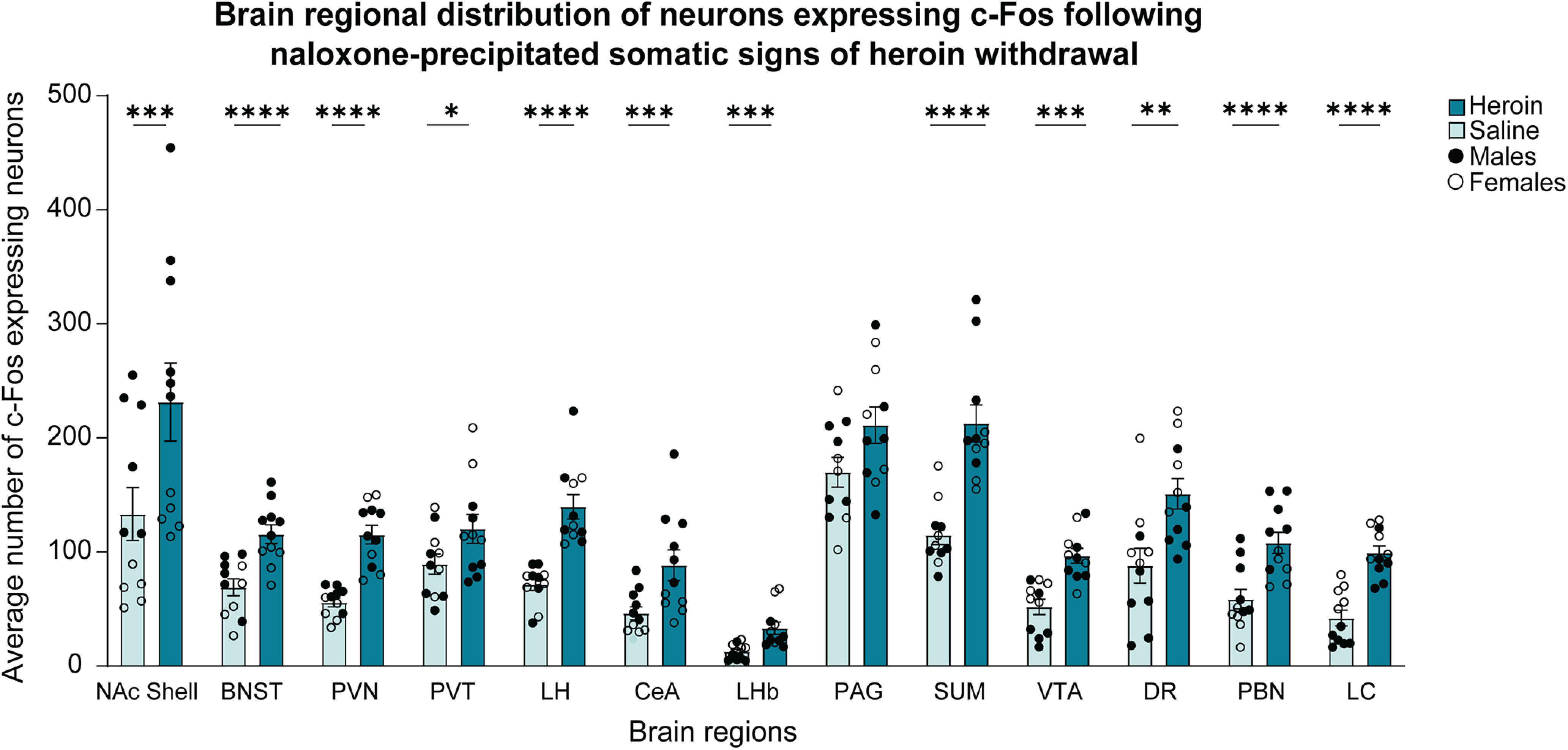
Regional distribution of c-Fos-expressing neurons following the assessment of naloxone-precipitated somatic signs of heroin withdrawal. Significant effects of treatment on c-Fos expression were found in the NAc shell (***p <* *0.001), BNST (****p <* *0.0001), PVN (****p <* *0.0001), PVT (*p <* *0.05), LH (****p <* *0.0001), CeA (***p <* *0.001), LHb (***p <* *0.001), SUM (****p <* *0.0001), VTA (***p <* *0.001), DR (**p <* *0.01), PBN (****p <* *0.0001), and LC (****p <* *0.0001). The data are expressed as mean ± SEM *n *=* *11/group (male and female data combined). A comparison of c-Fos expression levels between heroin-treated and saline-treated male and female mice is shown in Extended Data Figure 4-1.

To investigate brain region-specific activity that is associated with naloxone-precipitated withdrawal, we quantified c-Fos expression in brains that were collected 3 h after the last heroin injection (1 h after the naloxone injection; Fig. 2*A*,*B*). Statistical values are shown in Table 2. We found an increase in c-Fos expression in female mice compared with male mice in the PVT, LHb, DR, and LC and a decrease in c-Fos expression in female mice compared with male mice in the NAc shell, BNST, CeA, and PBN. The two-way ANOVAs for each brain region showed a significant sex × treatment interaction only for the SUM, but the *post hoc* comparisons did not reveal significant differences (Extended Data Fig. 4-1). The ANOVA detected significant effects of treatment (heroin > saline) on c-Fos expression in the NAc shell, BNST, PVN, PVT, LH, CeA, LHb, SUM, VTA, DR, PBN, and LC. No significant effects of treatment were found on c-Fos expression in the PAG (Fig. 4). These results indicated that naloxone-precipitated heroin withdrawal significantly increased c-Fos expression in distinct brain regions in a sex-independent manner.

**Table 2:** Two-way ANOVA results of Fos quantification in analyzed brain regions following naloxone-precipitated somatic signs of opioid withdrawal

Brain region	Treatment effect	Sex effect	Sex × treatment interaction
NAc shell	*F*_(1,18)_ = 15.91, *p < *0.001	*F*_(1,18)_ = 40.50, *p *<* *0.0001; M > F	*F*_(1,18)_ = 1.781, *p* = 0.1986
BNST	*F*_(1,18)_ = 29.53, *p < *0.0001	*F*_(1,18)_ = 14.97, *p* = 0.0011; M > F	*F*_(1,18)_ = 1.470, *p* = 0.2411
PVN	*F*_(1,18)_ = 43.11, *p < *0.0001	*F*_(1,18)_ = 1.915, *p* = 0.1833	*F*_(1,18)_ = 0.1469, *p* = 0.7060
PVT	*F*_(1,18)_ = 5.069, *p < *0.05	*F*_(1,18)_ = 4.738, *p* = 0.0431; F > M	*F*_(1,18)_ = 1.023, *p* = 0.3253
LH	*F*_(1,18)_ = 30.28, *p < *0.001	*F*_(1,18)_ = 0.3534, *p* = 0.5436	*F*_(1,18)_ = 0.04941, *p* = 0.8266
CeA	*F*_(1,18)_ = 17.40, *p < *0.001	*F*_(1,18)_ = 23.89, *p* = 0.0001; M > F	*F*_(1,18)_ = 3.703, *p* = 0.0703
LHb	*F*_(1,18)_ = 18.28, *p < *0.001	*F*_(1,18)_ = 8.314, *p* = 0.0099; F > M	*F*_(1,18)_ = 1.297, *p* = 0.2697
PAG	*F*_(1,18)_ = 3.855, *p = *0.0652	*F*_(1,18)_ = 0.02565, *p* = 0.8746	*F*_(1,18)_ = 0.3041, *p* = 0.5881
SUM	*F*_(1,18)_ = 33.96, *p < *0.0001	*F*_(1,18)_ = 1.128, *p* = 0.3023	*F*_(1,18)_ = 6.029, *p = *0.0245
VTA	*F*_(1,18)_ = 24.40, *p < *0.0001	*F*_(1,18)_ = 2.406, *p* = 0.1383	*F*_(1,18)_ = 1.791, *p* = 0.1975
DR	*F*_(1,18)_ = 14.54, *p < *0.01	*F*_(1,18)_ = 12.91, *p* = 0.0021; F > M	*F*_(1,18)_ = 0.116, *p* = 0.7422
PBN	*F*_(1,18)_ = 27.17, *p < *0.0001	*F*_(1,18)_ = 18.16, *p* = 0.0005; M > F	*F*_(1,18)_ = 0.1257, *p* = 0.7271
LC	*F*_(1,18)_ = 88.91, *p < *0.0001	*F*_(1,18)_ = 29.03, *p *<* *0.0001; F > M	*F*_(1,18)_ = 2.218, *p* = 0.1537

Males (M), Females (F).

10.1523/ENEURO.0106-22.2022.f4-1 Extended Data Figure 4-1 C-Fos expression in neurons distributed in distinct brain regions in male and female mice following the assessment of naloxone-precipitated somatic signs of heroin withdrawal. **B.** Two-way ANOVAs for each brain region showed a significant sex × treatment interaction only for the SUM (treatment effect: *F*_(1,18)_ = 33.96, *p < *0.0001; sex effect: *F*_(1,18)_ = 1.128, *p = *0.0245; sex × treatment interaction: *F*_(1,18)_ = 6.029, *p = *0.0245), but the *post hoc* comparisons did not detect meaningful differences (heroin females > saline males; heroin males > saline females). We found main treatment effect and main sex effect on the number of c-Fos-expressing neurons in several brain regions: NAc shell (treatment effect: *F*_(1,18)_ = 15.91, *p < *0.001; sex effect: *F*_(1,18)_ = 40.50, *p *<* *0.0001; sex × treatment interaction: *F*_(1,18)_ = 1.781, *p *=* *0.1986), BNST (treatment effect: *F*_(1,18)_ = 29.53, *p < *0.0001; sex effect: *F*_(1,18)_ = 14.97, *p *=* *0.0011; sex × treatment interaction: *F*_(1,18)_ = 1.470, *p *=* *0.2411), PVT (treatment effect: *F*_(1,18)_ = 5.069, *p < *0.05; sex effect: *F*_(1,18)_ = 4.738, *p *=* *0.0431; sex × treatment interaction: *F*_(1,18)_ = 1.023, *p *=* *0.3253), CeA (treatment effect: *F*_(1,18)_ = 17.40, *p < *0.001; sex effect: *F*_(1,18)_ = 23.89, *p *=* *0.0001; sex × treatment interaction: *F*_(1,18)_ = 3.703, *p *=* *0.0703), LHb (treatment effect: *F*_(1,18)_ = 18.28, *p < *0.001; sex effect: *F*_(1,18)_ = 8.314, *p *=* *0.0099; sex × treatment interaction: *F*_(1,18)_ = 1.297, *p *=* *0.2697), DR (treatment effect: *F*_(1,18)_ = 14.54, *p < *0.01; sex effect: *F*_(1,18)_ = 12.91, *p *=* *0.0021; sex × treatment interaction: *F*_(1,18)_ = 0.116, *p *=* *0.7422), PBN (treatment effect: *F*_(1,18)_ = 27.17, *p < *0.0001; sex effect: *F*_(1,18)_ = 18.16, *p *=* *0.0005; sex × treatment interaction: *F*_(1,18)_ = 0.1257, *p *=* *0.7271), and LC (treatment effect: *F*_(1,18)_ = 88.91, *p < *0.0001; sex effect: *F*_(1,18)_ = 29.03, *p *<* *0.0001; sex × treatment interaction: *F*_(1,18)_ = 2.218, *p *=* *0.1537). We found significant treatment effect but no sex effect in the following brain regions: PVN (treatment effect: *F*_(1,18)_ = 43.11, *p < *0.0001; sex effect: *F*_(1,18)_ = 1.915, *p *=* *0.1833; sex × treatment interaction: *F*_(1,18)_ = 0.1469, *p *=* *0.7060), LH (treatment effect: *F*_(1,18)_ = 30.28, *p < *0.001; sex effect: *F*_(1,18)_ = 0.3534, *p *=* *0.5436; sex × treatment interaction: *F*_(1,18)_ = 0.04941, *p *=* *0.8266), SUM (treatment effect: *F*_(1,18)_ = 33.96, *p < *0.0001; sex effect: *F*_(1,18)_ = 1.128, *p *=* *0.3023; sex × treatment interaction: *F*_(1,18)_ = 6.029, *p *=* *0.0245), and VTA (treatment effect: *F*_(1,18)_ = 24.40, *p < *0.0001; sex effect: *F*_(1,18)_ = 2.406, *p *=* *0.1383; sex × treatment interaction: *F*_(1,18)_ = 1.791, *p *=* *0.1975), We did not find a main treatment effect nor sex effect in the in the PAG (treatment effect: *F*_(1,18)_ = 3.855, *p = *0.0652; sex effect: *F*_(1,18)_ = 0.02565, *p *=* *0.8746; sex × treatment interaction: *F*_(1,18)_ = 0.3041, *p *=* *0.5881). Download Figure 4-1, TIF file.

### PCA of c-Fos expression and hyperalgesia during spontaneous withdrawal

**Figure 5.**
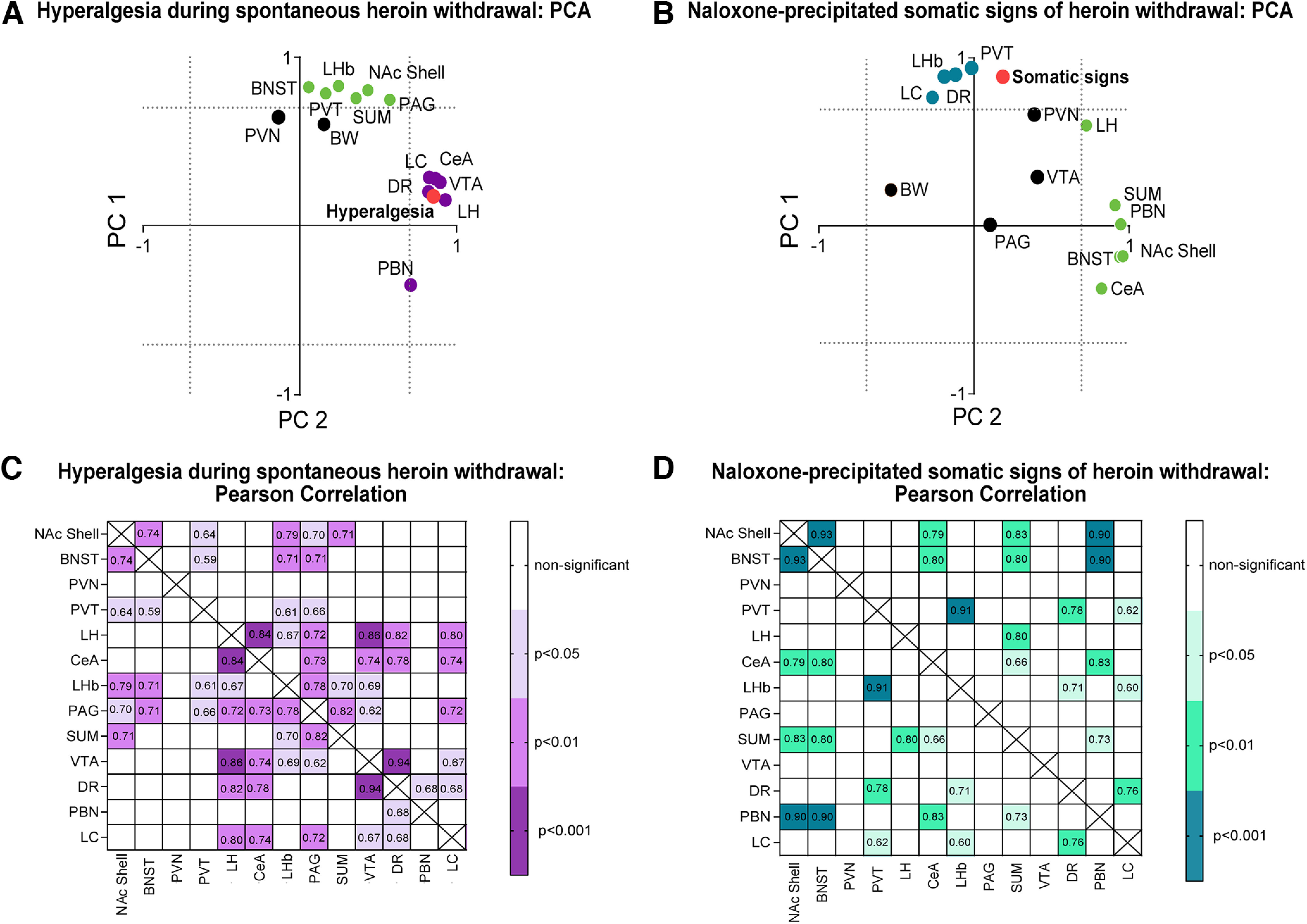
Hyperalgesia during spontaneous heroin withdrawal and naloxone-precipitated somatic signs of heroin withdrawal were associated with c-Fos expression in specific brain areas. ***A***, PCA of hyperalgesia and c-Fos expression in different brain regions in heroin-dependent mice. Hyperalgesia (red circle) and the DR, LC, CeA, VTA LH, and PBN (purple circles) loaded onto the PC1. The BNST, PVT, NAc shell, SUM, and PAG loaded onto the PC2 (green circles). Bodyweight loss (BW) and the PVN (black circles) loaded independently from PC1 and PC2. The distributions of variables are shown along the two factors (independent axes) with factor loadings ≥0.7 (indicated by dotted lines), produced by an orthogonal normalized varimax rotation. *n *=* *12/group (male and female data combined). ***B***, PCA of somatic withdrawal and Fos expression in different brain regions in heroin-dependent mice. The LH, SUM, PBN, BNST, NAc shell, and CeA (green circles) loaded onto PC1. Somatic withdrawal (red circle) and the DR, LC, LHb, and PVT (blue circles) loaded onto PC2. The BW, PVN, VTA, and PAG (black circles) loaded independently from PC1 and PC2. The distributions of variables are shown along the two factors with factor loadings ≥0.7, produced by an orthogonal normalized varimax rotation. *n *=* *11/group (male and female data combined). ***C***, Correlation of c-Fos expression in specific brain regions in heroin-dependent mice following the assessment of hyperalgesia during spontaneous withdrawal. Pearson correlation coefficients (*r*) inside the boxes correspond to regions that showed significant correlations (*p *<* *0.05). Darker purple shades represent stronger correlations. *n *=* *12/group (male and female data combined). ***D***, Correlations of c-Fos expression in specific brain regions in heroin-dependent mice following the assessment of naloxone-precipitated somatic signs of heroin withdrawal. Pearson correlation coefficients (*r*) inside the boxes correspond to regions that showed significant correlations (*p *<* *0.05). Darker green shades represent stronger correlations. *n *=* *11/group (male and female data combined).

To determine the relationship between neuronal activity and hyperalgesia during spontaneous heroin withdrawal, we performed PCA to reduce dimensionality of the data from heroin-treated mice. The PCA with an orthogonal normalized varimax rotation included the average number of c-Fos-expressing neurons of each brain region and Δ of paw withdrawal thresholds of male and female heroin-treated mice (Fig. 5*A*). The analysis showed two factors that together accounted for >70% of the data variance. Factor 1 accounted for 53.54% of the variance, whereas factor 2 accounted for 20.51% of the variance. Hyperalgesia and c-Fos expression in the DR, LC, CeA, VTA, LH, and PBN loaded onto the first principal component (PC1). The second principal component (PC2) included c-Fos expression in the BNST, PVT, LHb, NAc shell, SUM, and PAG. Because these factors were orthogonal to each other, we considered them independent from each other and inferred that brain regions that loaded onto PC1 may be functionally involved in hyperalgesia, whereas brain regions that loaded onto PC2 may be involved in spontaneous withdrawal signs other than hyperalgesia. Bodyweight loss and the PVN did not load onto PC1 or PC2.

### PCA of c-Fos expression and naloxone-precipitated somatic signs of opioid withdrawal

The PCA with an orthogonal normalized varimax rotation included the average number of c-Fos-expressing neurons of each brain region and somatic opioid withdrawal scores of male and female heroin-treated mice (Fig. 5*B*). The analysis showed two factors that together accounted for >70% of the data variance. The PC1 accounted for 37.03% of the variance and included the LH, SUM, PBN, BNST, NAc shell, and CeA. Somatic withdrawal and the DR, LC, LHb, and PVT loaded onto PC2 (accounting for 35.19% of the variance). From these findings, we inferred that brain regions that loaded onto PC2 may be involved in mediating somatic withdrawal, whereas brain regions that loaded onto PC1 may be associated with other aspects of precipitated withdrawal. Bodyweight loss, the PVN, VTA, and PAG did not load onto PC1 or PC2.

### Correlations of c-Fos expression among brain regions following hyperalgesia during spontaneous heroin withdrawal

We used Pearson’s correlation tests to correlate the average number of c-Fos-expressing neurons among the analyzed brain regions for male and female heroin-treated mice (Fig. 5*C*). Among the brain regions that loaded with hyperalgesia in the same principal component, we found that the LH and VTA were the only brain regions that correlated with the LHb (*r = *0.67, *p *<* *0.05, and *r = *0.69, *p *<* *0.05, respectively), and the PBN only correlated with the DR (*r = *0.68, *p *<* *0.05). Across brain regions, we observed the strongest correlations of c-Fos expression between the LH and CeA (*r = *0.84, *p *<* *0.001), between the LH and VTA (*r = *0.86, *p *<* *0.001), between the LH and DR (*r = *0.82, *p *<* *0.01), between the DR and VTA (*r = *0.94, *p < *0.001), and between the DR and CeA (*r = *0.78, *p *<* *0.01).

### Correlations of c-Fos expression among brain regions following naloxone-precipitated somatic signs of opioid withdrawal

Among the brain regions that loaded with somatic signs of opioid withdrawal in the same principal component, we found the strongest correlations of c-Fos expression between the PVT and LHb (*r = *0.91, *p *<* *0.001), between the DR and PVT (*r = *0.76, *p *<* *0.01), and between the DR and LC (*r = *0.76, *p *<* *0.01; Fig. 5*D*).

## Discussion

**Figure 6.**
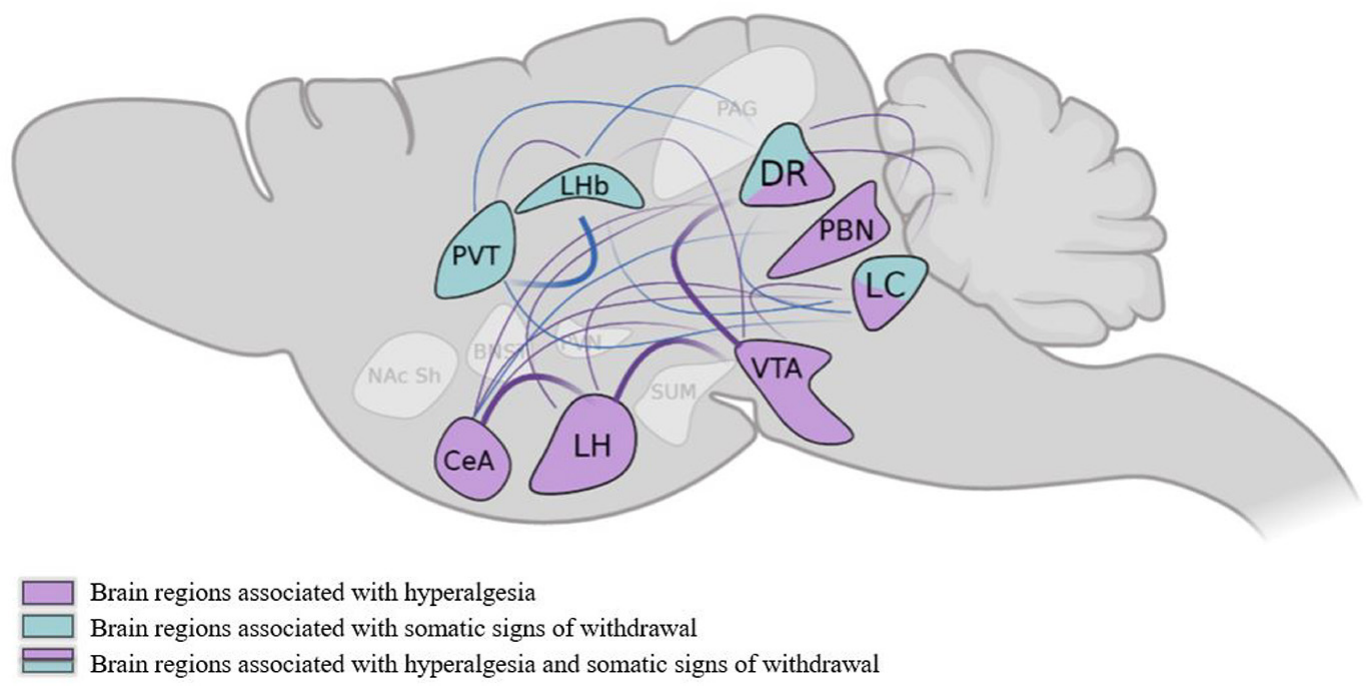
Summary of brain regions that are associated with neuronal c-Fos expression following the assessment of hyperalgesia during spontaneous withdrawal or naloxone-precipitated somatic signs of withdrawal in heroin-dependent mice. The colored brain regions were associated with PCA factors that loaded with hyperalgesia (purple), somatic signs of withdrawal (green), or both (purple/green) in heroin-dependent mice. The PBN, VTA, LH, and CeA are potentially involved in the expression of hyperalgesia during spontaneous withdrawal from heroin. The PVT and LHb are potentially involved in somatic signs of naloxone-precipitated heroin withdrawal. The DR and LC may play a role in both hyperalgesia and somatic signs of heroin withdrawal. The connecting lines represent significant correlations of c-Fos expression in brain regions that were associated with PCA factors that loaded with the behavior. The thickness of the lines represents the strength of correlations (thin = low correlation, *r < *0.84; thick = high correlation, *r > *0.84).

In the present study, we found associations between c-Fos expression and two aspects of opioid withdrawal. We showed that the administration of escalating doses of heroin caused hyperalgesia during spontaneous withdrawal and somatic signs of withdrawal that was precipitated by naloxone in male and female mice. We used higher doses of heroin in the naloxone-precipitated withdrawal experiment to obtain robust signs of somatic withdrawal to model observations in people during early stages of opioid detoxification. We measured hyperalgesia 16 h after spontaneous withdrawal to minimize the potential effect of somatic withdrawal signs at earlier withdrawal timepoints on von Frey testing. The automatic cell detection and counting of c-Fos, a marker of neuronal activity, indicated that hyperalgesia and somatic withdrawal were associated with c-Fos expression in various brain regions. No sex × treatment interactions were found in behavioral responses or c-Fos expression. The neuronal activity of some of these regions uniquely correlated with either hyperalgesia or somatic withdrawal, whereas c-Fos expression in the DR and LC correlated with both behaviors (summary in Fig. 6). Based on these findings, we suggest that selected brain networks participate in the expression of different opioid withdrawal signs.

Although some studies identified sex differences in hyperalgesia during heroin withdrawal in rats (Marchette et al., 2021), we did not find sex differences in the present study in mice. In addition to species differences and specific timepoints of behavior assessment, the lack of sex differences in the present study may have resulted from the use of relatively high doses of heroin in both male and female mice (40–50 mg/kg twice daily). For somatic withdrawal, the lack of sex differences in our study is consistent with previous studies in rats and mice (Cicero et al., 2002; Towers et al., 2019). Both distinct and similar cellular and molecular mechanisms have been linked to hyperalgesia and somatic withdrawal (Maldonado et al., 1996; Largent-Milnes et al., 2008; Tsai et al., 2009; Corder et al., 2013; Brust et al., 2015). For example, κ-opioid receptor antagonism reversed hyperalgesia during spontaneous heroin withdrawal in male and female rats but did not affect somatic withdrawal (Marchette et al., 2021). However, antagonists of NMDA glutamate receptors, β2-adrenergic receptors, serotonin 5-hydroxytryptamine-3 (5-HT_3_) receptors, and corticotropin-releasing factor 1 (CRF_1_) receptors attenuated both somatic withdrawal (Pinelli et al., 1997; Lu et al., 2000; Liang et al., 2007; Higgins et al., 2019) and hyperalgesia (Higgins et al., 1992; Liang et al., 2006, 2011; Harris et al., 2008; Park et al., 2015) in male rats and mice. These findings suggest both similarities and differences in the mechanisms that underlie hyperalgesia and somatic withdrawal.

We did not find sex × treatment interactions for c-Fos expression that was associated with hyperalgesia. For somatic withdrawal, although we found a significant sex × treatment interaction for c-Fos levels in the SUM, the *post hoc* comparisons did not indicate meaningful sex differences. Therefore, we combined the male and female data for subsequent analyses. We observed significant correlations between hyperalgesia during spontaneous heroin withdrawal and c-Fos expression in the LH, CeA, VTA, DR, PBN, and LC. Previous studies reported increases in c-Fos expression in the CeA and LC during naloxone withdrawal-induced hyperalgesia in male and female oxycodone-dependent rats (Simpson et al., 2020). Consistent with the role of the CeA in mediating the neuropharmacological effect of heroin, an increase in BOLD activity was detected in amygdala nuclei during the presentation of cues that were associated with naloxone-induced heroin self-administration (Carmack et al., 2019). Additionally, pharmacological studies found that the intra-CeA administration of a CRF receptor antagonist reduced naloxone-precipitated hyperalgesia in male morphine-dependent rats (McNally and Akil, 2002). Studies that focused on the DR showed the local activation of microglia and astrocytes in male fentanyl-dependent rats, and the systemic administration of an anti-inflammatory agent prevented fentanyl withdrawal-induced hyperalgesia (Carranza-Aguilar et al., 2022). Another study demonstrated that μ-opioid receptor knock-out in the DR prevented the heroin withdrawal-induced reduction of social interaction in mice (Lutz et al., 2014).

We found a correlation between naloxone-precipitated somatic signs of withdrawal and c-Fos expression in some brain areas that were previously associated with opioid withdrawal, such as the DR, LC, LHb, and PVT. Studies of the LC found local increases in neuronal firing rate and upregulation of the cyclic adenosine monophosphate pathway during the expression of somatic signs of withdrawal in male rats (Rasmussen et al., 1990). Pharmacological studies of the LC also showed that intra-LC glutamate or norepinephrine neurotransmission blockers attenuated somatic signs of opioid withdrawal (Taylor et al., 1988, 1998). Neuronal circuitry studies established that excitatory projections from the PVT to the medial shell of the NAc are necessary for the mediation of somatic withdrawal in male mice (Zhu et al., 2016). Other brain areas where we detected a positive correlation of c-Fos expression are the LHb and DR, which have been previously implicated in morphine withdrawal (Valentinova et al., 2019).

Among the different brain areas that we analyzed, we found that c-Fos expression in the LC and DR correlated with both hyperalgesia during spontaneous withdrawal and somatic signs of naloxone-precipitated withdrawal. The LC is a primary noradrenergic nucleus that controls autonomic function and the stress response (Chandler, 2016). The DR contains the largest population of neurons that release serotonin in the forebrain and midbrain regions (Steinbusch, 1981). Studies of global alterations of the noradrenergic system by lesioning catecholamine neurons in the LC demonstrated that this global manipulation does not alter naloxone-induced conditioned place aversion, the expression of somatic withdrawal, or the attenuation of somatic withdrawal by the α_2_-adrenergic agonist clonidine (Britton et al., 1984; Caillé et al., 1999). In contrast to global manipulation of the noradrenergic system, the intra-LC administration of clonidine attenuated behavioral and neurochemical changes that were associated with naloxone-precipitated withdrawal (Taylor et al., 1988). Studies of the DR showed that the global lesion of serotonergic neurons (Caillé et al., 2002) and μ-opioid receptor knock-out in the DR (Lutz et al., 2014) did not affect somatic signs of withdrawal. However, the systemic administration of a serotonin 5-HT_2A_ receptor antagonist attenuated somatic signs of naloxone-precipitated heroin withdrawal (Bing et al., 2022). Other behavioral studies found that norepinephrine transmission and impairments in serotonergic signaling are associated with motivational and somatic withdrawal in opioid-dependent animals (Goeldner et al., 2011; Koob, 2021). Altogether, these findings suggest that the activation of specific types of neurons (via specific connectivity) in the LC and DR play distinct roles in different aspects of opioid withdrawal and highlight the complexity of brain network activity in mediating opioid withdrawal.

We found positive correlations among c-Fos levels in brain regions that are associated with hyperalgesia and somatic signs of withdrawal (see summary in Fig. 6). For c-Fos levels in brain regions that are associated with hyperalgesia, we detected a positive correlation between the LH and the CeA and VTA. Projections from the CeA to the LH mediate the avoidance of a stress-associated stimulus (i.e., a predictor of a poor response to substance use treatment; Weera et al., 2021). Moreover, a recent circuit-based study demonstrated that a LH projection to the VTA was associated with reward-omitted outcomes and defensive response to threatening stimuli (de Jong et al., 2019; Barbano et al., 2020). Positive correlations were also found between the DR and CeA. Projections from the DR to the CeA have been implicated in compulsive-like cocaine taking (Verheij et al., 2018). For c-Fos levels in brain regions that are associated with somatic withdrawal, we detected a positive correlation of c-Fos expression between the LHb and DR. Network connectivity studies reported that the inactivation of LHb glutamatergic projections to the DR reversed the reduction of sociability during morphine withdrawal in mice (Valentinova et al., 2019). Altogether, these results suggest a complex network of brain regions that could participate in the expression of hyperalgesia and somatic signs of opioid withdrawal.

We found no association between bodyweight loss during heroin treatment and hyperalgesia or somatic signs of withdrawal. This suggests that bodyweight loss is not a predictor for either of these two behavioral measures. Although we found an increase in c-Fos expression in the NAc shell, BNST, PVN, SUM, and PAG, none of these brain regions correlated with hyperalgesia during spontaneous withdrawal or somatic signs of naloxone-precipitated withdrawal. The activation of these brain regions may mediate motivational opioid withdrawal-related behaviors that were not evaluated in the present study (e.g., aversion, anhedonia, despair-like behavior, social deficits, and anxiety-like behavior). Microinjections of a β-adrenergic receptor blocker and α_2_-adrenergic receptor agonist in the BNST (Delfs et al., 2000) and an AMPA receptor antagonist in the NAc shell (Russell et al., 2016) prevented naloxone-induced conditioned place aversion but had no effect on somatic withdrawal in male morphine-dependent rats. Studies of the NAc shell, BNST, PVN, SUM, and PAG may provide information about the roles of these brain regions in other opioid-related phenomena.

Clonidine and lofexidine, α_2_-adrenergic receptor agonists that suppress the adrenergic system, are used to treat somatic withdrawal symptoms during the acute phase of opioid withdrawal in humans, but they are ineffective for the long-term treatment of OUD (Gowing et al., 2009). Methadone and buprenorphine suppress both somatic and motivational symptoms and are used to treat OUD because they maintain opioid dependence. However, these medications have side-effects, and their discontinuation leads to withdrawal (Amato et al., 2004). Naltrexone decreases craving in people with OUD, but it is rarely prescribed because treatment initiation requires opioid abstinence, and poor adherence to treatment is an issue (Minozzi et al., 2011). The discovery of nonopioid neurotransmitter systems that mediate somatic and motivational symptoms of withdrawal could improve treatment outcomes for individuals with OUD. Thus, understanding common mechanisms that underlie somatic and motivational withdrawal could lead to new targets for drug development for OUD.

The present study has some limitations. We did not have groups of mice that received an acute injection of saline to make comparisons with the acute naloxone injection. However, the low c-Fos levels in chronically saline-treated mice compared with chronically heroin-treated mice following an acute injection of naloxone suggest that naloxone played a minimal role in c-Fos expression in nondependent mice. Although we did not design our experiments to directly compare c-Fos expression following the assessment of somatic withdrawal and hyperalgesia, c-Fos levels appeared to be lower in chronic saline-treated mice that received an acute naloxone injection than in chronic saline-treated mice that were tested for mechanical hyperalgesia. These results suggest that tactile stimulation of the hind paw with the rigid von Frey filament contributed to c-Fos expression in chronically saline-treated mice. Finally, we used passive injections of high heroin doses, so caution is needed when extrapolating our findings to lower doses or active drug intake.

In conclusion, hyperalgesia during spontaneous heroin withdrawal and somatic withdrawal were associated with the activity of different autonomic and limbic brain regions. Activity in the DR and LC, reflected by c-Fos expression, was associated with both behaviors in male and female mice. Future region-specific and cell type-specific studies will determine the functional involvement of different brain regions and neurotransmitter systems in negative emotional states (hyperkatifeia) that are associated with opioid withdrawal. Understanding alterations of neuronal activity and identifying potential neuronal networks that are associated with opioid withdrawal-related behaviors will also guide efforts to identify biomarkers of OUD and relapse.

10.1523/ENEURO.0106-22.2022.ed Extended Data Macros used for preprocessing and for morphological segmentation of images using Image-Pro 10.0 software. Download Extended Data 1, DOCX file.

## Synthesis

Reviewing Editor: Tod E Kippin, University of California Berkeley Decisions are customarily a result of the Reviewing Editor and the peer reviewers coming together and discussing their recommendations until a consensus is reached. When revisions are invited, a fact-based synthesis statement explaining their decision and outlining what is needed to prepare a revision will be listed below. The following reviewer(s) agreed to reveal their identity: Joel Schlosburg.

I have received two independent reviews of your manuscript, both of which are highly positive about your study, but also indicate the need for revision in order for it to be suitable for publication in eNeuro. Overall, the reviewers found the work to be an impressively comprehensive and thorough study expected to advance the field. However, each had concerns regarding specifics of the statistical approach as well as inclusion in main body or interpretations of the findings. As such, I encourage you to revise your manuscript after addressing each of the specific concerns raised by the reviewers. Again, both feel this study has the potential to impact the field.

Reviewer 1

The authors investigated spontaneous and precipitated heroin withdrawal in mice using hyperalgesia measurements (von Frey testing) and somatic withdrawal signs and evaluated the impact on recruitment of neuronal ensembles using Fos immunohistochemistry in 13 subcortical brain regions. They show that precipitated and spontaneous withdrawal recruits an overlapping network of brain regions (LH, CeA, LHb, VTA, DR, PBN, LC). Precipitated recruited additional brain regions including the NAc shell, BNST, PVT, and SUM. Principal component analysis revealed that hyperalgesia measures clustered with fos expression in the LC, DR, CeA, VTA, PBN and LH on the first principal component, while during precipitated withdrawal, somatic signs clustered with LC, DR, PVT and LHb on the second principal component. This is an interesting finding. The paper is well written, the methods are sound, and the results are clear-cut. However, the paper falls a little short and could be significantly improved, by adding additional results, restructuring the results and strengthening the discussion.

Strengths:

Comparison of spontaneous and precipitated withdrawal

Large number of brain regions analyzed in an unbiased way

Males and females are included

Weaknesses:

Lack of cortical brain regions

Additional data analysis may be needed

Major concerns

1. A lot of highly relevant results are in supplementary data. I highly recommend moving most of the supplementary data figures should be in the main text. For instance male vs female bar graphs, weight data (S2), scoring of the individual behaviors somatic signs (S3), raw data from the von frey testing (S1).

2. Since the experimental design was slightly different between the two experiments, dose regimen (precipitated withdrawal used higher dose), timing, it is unclear if the difference in Fos activation is due to the dose, timing, or the fact that it is precipitated by naloxone or because the behavioral assay was different (von frey, somatic signs). While I agree that these small differences may not be the main cause of the difference in Fos activation, it remains a possibility. The author should discuss this point.

3. Loss of bodyweight was measured for the spontaneous animals, I am curious to see how it relates to the other factors in the PCA (this could be added as supplemental results).

4. The lack of saline control for the naltrexone injection makes it a bit more difficult to interpret the data as it is possible that the stress of the injection contributed to the effects observed. This is unlikely as it appears that the Fos levels in the saline-naloxone group are relatively low but it should be discussed.

5. Related to the previous point, there is a large difference between the saline groups in the two experiments with Fos levels being sometimes 2-3x lower during the precipitated withdrawal paradigm. These differences should be further discussed and interpreted.

6. The authors repeatedly claim that there is no sex difference in this study. While there is only 1 sex*treatment interaction (SUM), and I agree that there are no major sex by treatment effects in this study, it appears that there are major sex differences in baseline Fos activity between brain regions that are known to be important for withdrawal effects. These effects should be interpreted and discussed, and the wording should be clarified. All sentences mentioning the “lack of sex difference” should be reworded to “lack of sex*treatment difference”.

7. It would be very useful to provide correlations between brain regions and behaviors to identify fos activity in brain regions that are the most predictive of the behavior.

8. In addition to the PCA/correlation analysis a multiple linear regression analysis would strengthen the results and perhaps identify the most critical regions for the behavior. Moreover, using this approach sex can be included as a variable to control for the effect of sex.

7. The correlation analysis was not really interpreted and discussed, but there might be some very relevant information in there. Correlation of the changes in Fos expression can be used as a measure of functional connectivity to build networks that can be used to identify the most important nodes and the flow of information in the network. Graph theory analysis or at least a visual representation of the network using edges thickness representing the strength of the correlations between regions would be very informative.

Minor concerns

1. Fig 3A and B: add additional ticks on the axis, potentially indicate with a dotted line the 0.7 cutoff for loading factors to be considered, this will make interpretation of the retained brain regions easier.

2. The statistical analysis needs improvement:

a. Please provide details on the animal numbers in the main manuscript and reasons for exclusion. Currently numbers are only given in the supplement and e.g. Fig S1 and Fig S2 are on the same animals but N = 23-28/group and N = 20/group (but yet 22 data points seem shown)

b. Check degrees of freedom e.g. Fig S2: 84 take into account baseline weight? Or Fig S3: 36 for interaction in number of jumps

c. Additional information regarding inclusion/exclusion of potential outliers should be provided, or whether such analysis was not performed.

3. The code of the macros used for quantifications should be provided as supplemental data or on an open source repository (github)

Reviewer 2

This manuscript is a fairly straightforward and comprehensive approach to using simple behavioral endpoints and correlating with basic neural activity. While the results may not be considered by some to be “groundbreaking", there is definitely something to be said for the workmanlike and thoroughness of the breadth of data collected for these studies. My only concerns or cautions would be in the language or discussion of the results.

1) I would be careful to make distinctions between withdrawal and opioid use disorder (which authors do a bit in discussion), as well as the approach of high-dose passive opioid induction as a model (and the shortcomings as a result).

2) Fos has some fairly short timelines of activation, and while the precipitated withdrawal has a fairly controlled time course, the hyperalgesia is taken at a particular time when clearly the “symptom” manifests. However, 16h may seem to some an arbitrary time point to choose and may or may not reflect aspects of neuronal activation that lead to the behavioral manifestation. Perhaps reduced MOR expression or endogenous opioids were genetically encoded a day prior. Rather than doing a monumental time course experiment, at least the authors can discuss the rationale of the time point chosen and the limitations of their “snapshot” in time.
