# Supplementary figures and images for: Neuronal Correlates of Hyperalgesia and Somatic Signs of Heroin Withdrawal in Male and Female Mice
Source: eNeuro. 2022 Jul 6;9(4):ENEURO.0106-22.2022. doi: 10.1523/ENEURO.0106-22.2022 (PMC9267003; doi:10.1523/ENEURO.0106-22.2022)

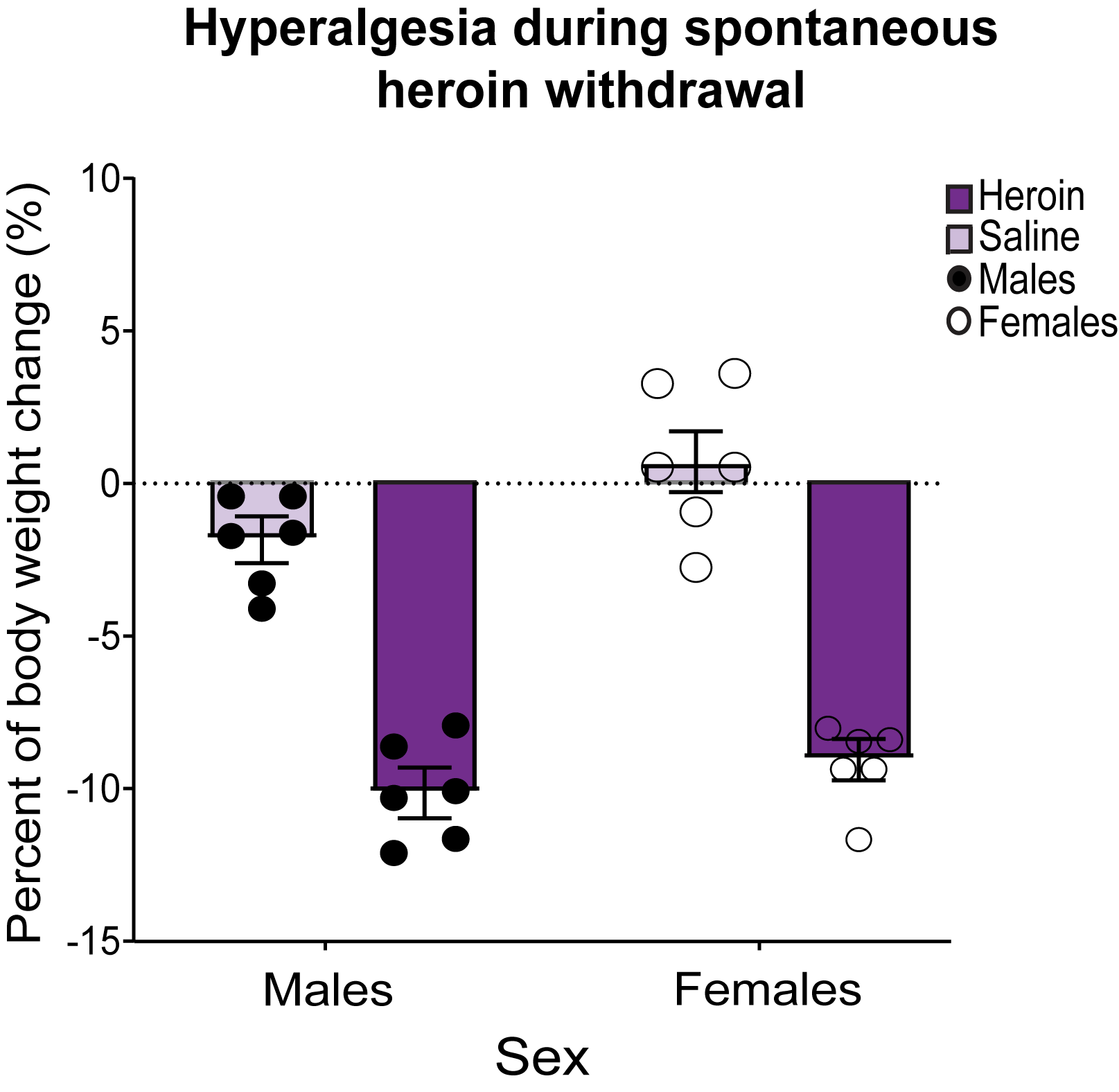

Supplement: Extended Data Figure 1-1 — Repeated heroin injections caused bodyweight loss in male and female mice. Percent of bodyweight change in mice that were used for the c-Fos expression experiment following the assessment of hyperalgesia during spontaneous heroin withdrawal. The Student’s t test showed that heroin-treated mice (male and female data combined) weighed significantly less than saline-treated mice (****p < 0.0001). The data are expressed as mean ± SEM. N = 12/group. Download Figure 1-1, TIF file. [file enu-eN-NWR-0106-22-s02.tif]

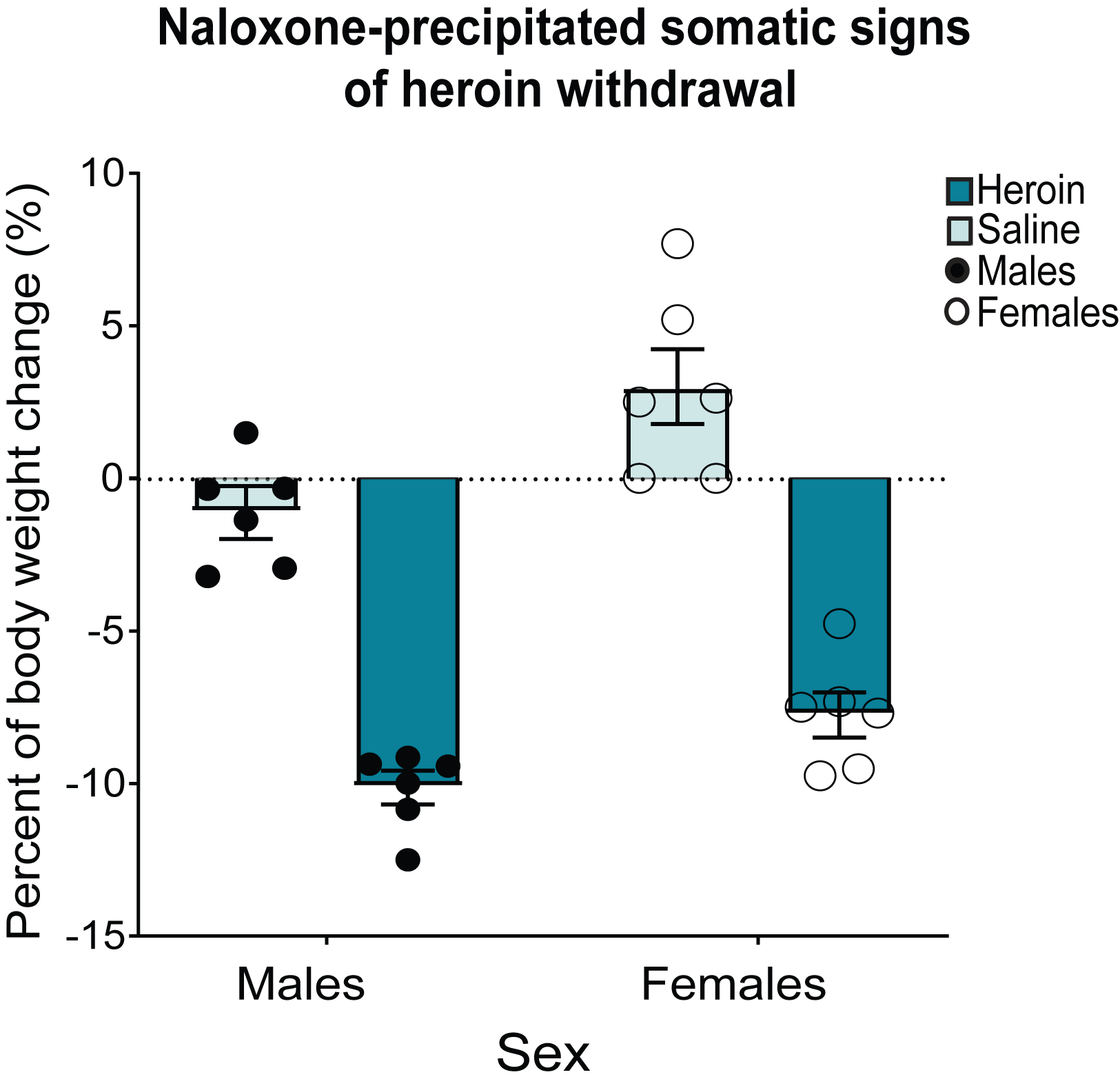

Supplement: Extended Data Figure 2-1 — Repeated heroin injections caused bodyweight loss in male and female mice. Percent of bodyweight change in mice used for the c-Fos expression experiment following the assessment of naloxone-precipitated somatic signs of opioid withdrawal. The Student’s t test showed that heroin-treated mice (male and female data combined) weighed significantly less than saline mice (****p < 0.0001). The data are expressed as mean ± SEM. N = 12/group. Download Figure 2-1, TIF file. [file enu-eN-NWR-0106-22-s03.tif]

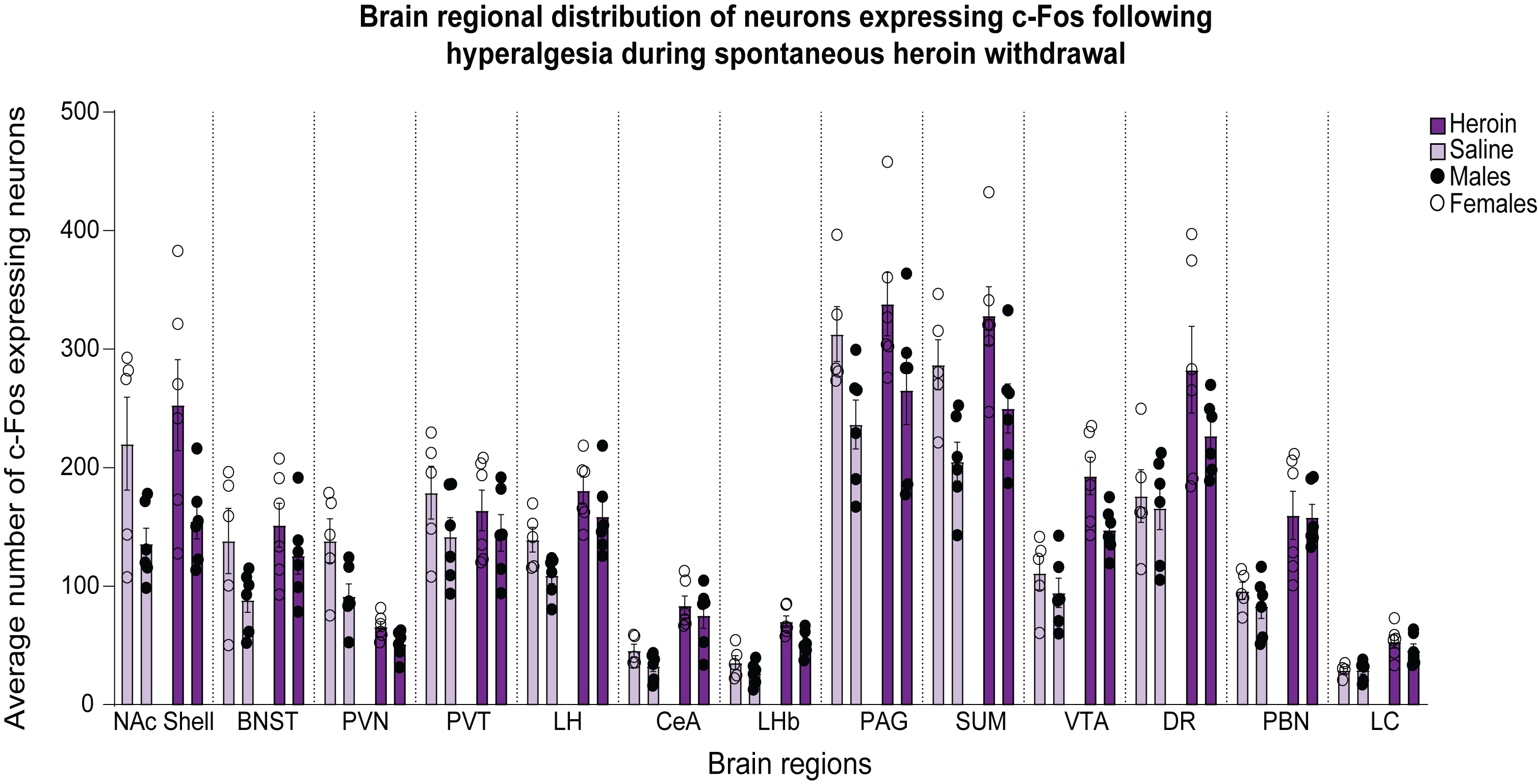

Supplement: Extended Data Figure 3-1 — C-Fos expression in neurons distributed in distinct brain regions in male and female mice following the assessment of hyperalgesia during spontaneous heroin withdrawal. Two-way ANOVAs showed no sex × treatment interactions for any of the analyzed brain regions. We found main treatment effect and main sex effect on the number c-Fos-expressing neurons in several brain regions: LH (treatment effect: F(1,19) = 16.93, p < 0.001; sex effect: F(1,19) = 5.546, p = 0.0294; sex × treatment interaction: F(1,19) = 0.1327, p = 0.7197), LHb (treatment effect: F(1,19) = 40.80, p < 0.0001; sex effect: F(1,19) = 8.195, p = 0.0100; sex × treatment interaction: F(1,19) = 0.9071, p = 0.3528), VTA (treatment effect: F(1,19) = 27.39, p < 0.0001; sex effect: F(1,19) = 5.773, p = 0.0267; sex × treatment interaction: F(1,19) = 0.1231, p = 0.2810), PVN (treatment effect: F(1,19) = 30.16, p < 0.0001; sex effect: F(1,19) = 8.964, p = 0.0075; sex × treatment interaction: F(1,19) = 0.2445, p = 0.1344), and SUM (treatment effect: F(1,19) = 4.195, p = 0.0546; sex effect: F(1,19) = 14.44, p = 0.0012; sex × treatment interaction: F(1,19) = 0.007174, p = 0.9334). We found significant treatment effect but no sex effect in the following brain regions: CeA (treatment effect: F(1,19) = 26.37, p < 0.0001; sex effect: F(1,19) = 1.876, p = 0.1867; sex × treatment interaction: F(1,19) = 0.09373, p = 0.7628), DR (treatment effect: F(1,19) = 11.70, p < 0.01; sex effect: F(1,19) = 1.803, p = 0.1952; sex × treatment interaction: F(1,19) = 0.08641, p = 0.3642), PBN (treatment effect: F(1,19) = 26.15, p < 0.0001; sex effect: F(1,19) = 0.2822, p = 0.6014; sex × treatment interaction: F(1,19) = 0.1759, p = 0.6796), and LC (treatment effect: F(1,19) = 17.91, p < 0.001; sex effect: F(1,19) = 0.5460, p = 0.4695; sex × treatment interaction: F(1,19) = 0.5909, p = 0.4520). We did not find significant treatment effects, but we found main sex effects on the number of c-Fos-expressing neurons in three brain regions: NAc she [file enu-eN-NWR-0106-22-s04.tif]

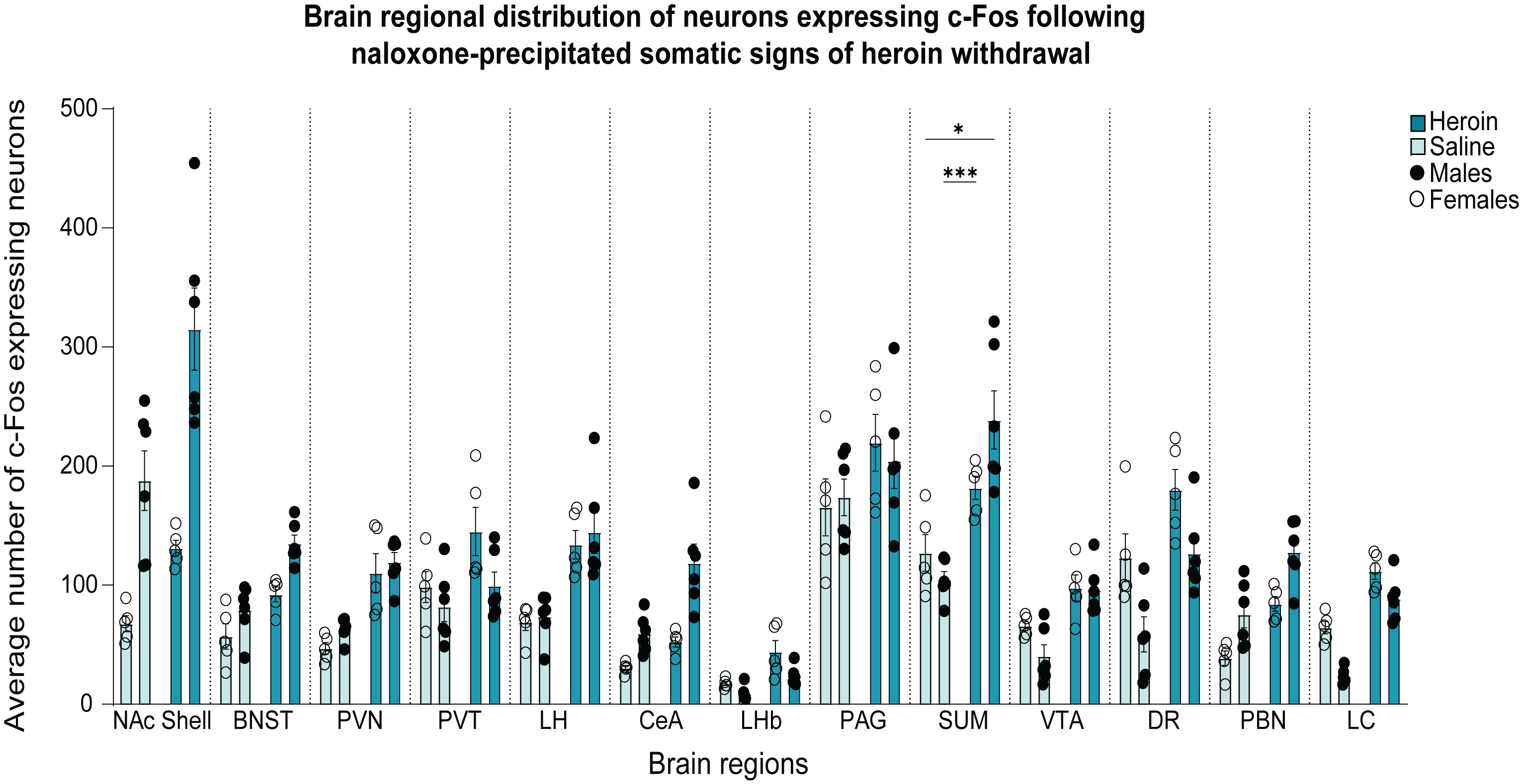

Supplement: Extended Data Figure 4-1 — C-Fos expression in neurons distributed in distinct brain regions in male and female mice following the assessment of naloxone-precipitated somatic signs of heroin withdrawal. B. Two-way ANOVAs for each brain region showed a significant sex × treatment interaction only for the SUM (treatment effect: F(1,18) = 33.96, p < 0.0001; sex effect: F(1,18) = 1.128, p = 0.0245; sex × treatment interaction: F(1,18) = 6.029, p = 0.0245), but the post hoc comparisons did not detect meaningful differences (heroin females > saline males; heroin males > saline females). We found main treatment effect and main sex effect on the number of c-Fos-expressing neurons in several brain regions: NAc shell (treatment effect: F(1,18) = 15.91, p < 0.001; sex effect: F(1,18) = 40.50, p < 0.0001; sex × treatment interaction: F(1,18) = 1.781, p = 0.1986), BNST (treatment effect: F(1,18) = 29.53, p < 0.0001; sex effect: F(1,18) = 14.97, p = 0.0011; sex × treatment interaction: F(1,18) = 1.470, p = 0.2411), PVT (treatment effect: F(1,18) = 5.069, p < 0.05; sex effect: F(1,18) = 4.738, p = 0.0431; sex × treatment interaction: F(1,18) = 1.023, p = 0.3253), CeA (treatment effect: F(1,18) = 17.40, p < 0.001; sex effect: F(1,18) = 23.89, p = 0.0001; sex × treatment interaction: F(1,18) = 3.703, p = 0.0703), LHb (treatment effect: F(1,18) = 18.28, p < 0.001; sex effect: F(1,18) = 8.314, p = 0.0099; sex × treatment interaction: F(1,18) = 1.297, p = 0.2697), DR (treatment effect: F(1,18) = 14.54, p < 0.01; sex effect: F(1,18) = 12.91, p = 0.0021; sex × treatment interaction: F(1,18) = 0.116, p = 0.7422), PBN (treatment effect: F(1,18) = 27.17, p < 0.0001; sex effect: F(1,18) = 18.16, p = 0.0005; sex × treatment interaction: F(1,18) = 0.1257, p = 0.7271), and LC (treatment effect: F(1,18) = 88.91, p < 0.0001; sex effect: F(1,18) = 29.03, p < 0.0001; sex × treatment interaction: F(1,18) = 2.218, p = 0.1537). We found significant treatment effect but no sex effect in the following brain regions: PVN (treatmen [file enu-eN-NWR-0106-22-s05.tif]
