# Supplementary material for: Neuronal Correlates of Hyperalgesia and Somatic Signs of Heroin Withdrawal in Male and Female Mice
Source: eNeuro. 2022 Jul 6;9(4):ENEURO.0106-22.2022. doi: 10.1523/ENEURO.0106-22.2022 (PMC9267003; doi:10.1523/ENEURO.0106-22.2022)
Supplement: Extended Data — Macros used for preprocessing and for morphological segmentation of images using Image-Pro 10.0 software. Download Extended Data 1, DOCX file. [file enu-eN-NWR-0106-22-s06.docx]

**Extended Data 1. Macros used for pre-processing and for morphological segmentation of images using Image-Pro 10.0 software.**

**Macro for Pre-Processing Images**

Public Function PreProYAB_Calibration__um___IN_SITU_20XTiffVS_Naming_Saving_Copy() As SimpleScript

PreProYAB_Calibration__um___IN_SITU_20XTiffVS_Naming_Saving_Copy = New SimpleScript

Dim var1 = "Spatial Cal In situ Focus 20x", spcal1, doc1, image1, doc2, image2, doc3, image3, doc4, image4, image5, doc5, image6, window1, window2, window3, window4, varList1

With Application.RibbonCommands.SelectRibbonTab(PreProYAB_Calibration__um___IN_SITU_20XTiffVS_Naming_Saving_Copy)

.TabName = "Home"

.Run()

End With

With Measure.Calibration.SpatialCommands.Define(PreProYAB_Calibration__um___IN_SITU_20XTiffVS_Naming_Saving_Copy)

.Run(var1, spcal1)

End With

With Measure.Calibration.SpatialCommands.SetActive(PreProYAB_Calibration__um___IN_SITU_20XTiffVS_Naming_Saving_Copy)

.Comment = "Spatial Cal In situ Focus 20x: 0.3437 x 0.3437 µm"

.FilterInput = True

.Run(spcal1)

End With

With Application.DocumentCommands.Active(PreProYAB_Calibration__um___IN_SITU_20XTiffVS_Naming_Saving_Copy)

.Run(doc1)

End With

With Measure.Calibration.SpatialCommands.Apply(PreProYAB_Calibration__um___IN_SITU_20XTiffVS_Naming_Saving_Copy)

.Comment = "Spatial Cal In situ Focus 20x: 0.3437 x 0.3437 µm"

.Run(doc1, spcal1)

End With

With Application.RibbonCommands.SelectRibbonTab(PreProYAB_Calibration__um___IN_SITU_20XTiffVS_Naming_Saving_Copy)

.TabName = "Adjust"

.Run()

End With

With Adjust.ImageCommands.Rotate(PreProYAB_Calibration__um___IN_SITU_20XTiffVS_Naming_Saving_Copy)

.Orient = Image.OrientType.Rotate180

.Visible = True

.Run(doc1, image1)

End With

With Application.DocumentCommands.Activate(PreProYAB_Calibration__um___IN_SITU_20XTiffVS_Naming_Saving_Copy)

.Run(image1, doc2)

End With

With Adjust.ImageCommands.ExtractChannel(PreProYAB_Calibration__um___IN_SITU_20XTiffVS_Naming_Saving_Copy)

.Channel = 2

.Interpretation = MediaCy.IQL.Engine.mcInterpretation.mciRGB

.Run(image1, image2)

End With

With Application.DocumentCommands.Activate(PreProYAB_Calibration__um___IN_SITU_20XTiffVS_Naming_Saving_Copy)

.Run(image2, doc3)

End With

With Application.RibbonCommands.SelectRibbonTab(PreProYAB_Calibration__um___IN_SITU_20XTiffVS_Naming_Saving_Copy)

.TabName = "Process"

.Run()

End With

With Adjust.ImageCommands.Duplicate(PreProYAB_Calibration__um___IN_SITU_20XTiffVS_Naming_Saving_Copy)

.Name = "Layer 1_Rotate180CW_Blue_BGSubtracted"

.Visible = True

.Run(doc3, image3)

End With

With Application.DocumentCommands.Activate(PreProYAB_Calibration__um___IN_SITU_20XTiffVS_Naming_Saving_Copy)

.Run(image3, doc4)

End With

With Process.BackgroundCommands.Extract(PreProYAB_Calibration__um___IN_SITU_20XTiffVS_Naming_Saving_Copy)

.BackgroundType = Background.ExtractTypes.Bright

.FeatureSize = 20

.Visible = False

.Run(doc4, image4)

End With

With Process.BackgroundCommands.Subtract(PreProYAB_Calibration__um___IN_SITU_20XTiffVS_Naming_Saving_Copy)

.Run(doc4, image4, image3)

End With

With Adjust.ImageCommands.Duplicate(PreProYAB_Calibration__um___IN_SITU_20XTiffVS_Naming_Saving_Copy)

.Name = "Layer 1_Rotate180CW_Blue_BGSubtracted_BGCorrected"

.Visible = True

.Run(doc4, image5)

End With

With Application.DocumentCommands.Activate(PreProYAB_Calibration__um___IN_SITU_20XTiffVS_Naming_Saving_Copy)

.Run(image5, doc5)

End With

With Process.BackgroundCommands.Extract(PreProYAB_Calibration__um___IN_SITU_20XTiffVS_Naming_Saving_Copy)

.BackgroundType = Background.ExtractTypes.Bright

.FeatureSize = 20

.Visible = False

.Run(doc5, image6)

End With

With Process.BackgroundCommands.Correct(PreProYAB_Calibration__um___IN_SITU_20XTiffVS_Naming_Saving_Copy)

.BlackLevel = 0R

.Run(doc5, image6, image5)

End With

With Application.DocumentCommands.Activate(PreProYAB_Calibration__um___IN_SITU_20XTiffVS_Naming_Saving_Copy)

.Run(doc1, doc1)

End With

With Application.DocumentCommands.Activate(PreProYAB_Calibration__um___IN_SITU_20XTiffVS_Naming_Saving_Copy)

.Run(doc5, doc5)

End With

With Application.DocumentCommands.Properties(PreProYAB_Calibration__um___IN_SITU_20XTiffVS_Naming_Saving_Copy)

.DisplayName = "Layer .tif"

.Run(doc5)

End With

With Application.DocumentCommands.Activate(PreProYAB_Calibration__um___IN_SITU_20XTiffVS_Naming_Saving_Copy)

.Run(doc4, doc4)

End With

With Application.WindowCommands.Define(PreProYAB_Calibration__um___IN_SITU_20XTiffVS_Naming_Saving_Copy)

.Run(doc4, window1)

End With

With Application.WindowCommands.Close(PreProYAB_Calibration__um___IN_SITU_20XTiffVS_Naming_Saving_Copy)

.Run(window1)

End With

With Application.DocumentCommands.Activate(PreProYAB_Calibration__um___IN_SITU_20XTiffVS_Naming_Saving_Copy)

.Run(doc5, doc5)

End With

With Application.DocumentCommands.Activate(PreProYAB_Calibration__um___IN_SITU_20XTiffVS_Naming_Saving_Copy)

.Run(doc3, doc3)

End With

With Application.WindowCommands.Define(PreProYAB_Calibration__um___IN_SITU_20XTiffVS_Naming_Saving_Copy)

.Run(doc3, window2)

End With

With Application.WindowCommands.Close(PreProYAB_Calibration__um___IN_SITU_20XTiffVS_Naming_Saving_Copy)

.Run(window2)

End With

With Application.DocumentCommands.Activate(PreProYAB_Calibration__um___IN_SITU_20XTiffVS_Naming_Saving_Copy)

.Run(doc5, doc5)

End With

With Application.DocumentCommands.Activate(PreProYAB_Calibration__um___IN_SITU_20XTiffVS_Naming_Saving_Copy)

.Run(doc2, doc2)

End With

With Application.WindowCommands.Define(PreProYAB_Calibration__um___IN_SITU_20XTiffVS_Naming_Saving_Copy)

.Run(doc2, window3)

End With

With Application.WindowCommands.Close(PreProYAB_Calibration__um___IN_SITU_20XTiffVS_Naming_Saving_Copy)

.Run(window3)

End With

With Application.DocumentCommands.Activate(PreProYAB_Calibration__um___IN_SITU_20XTiffVS_Naming_Saving_Copy)

.Run(doc5, doc5)

End With

With Application.DocumentCommands.Activate(PreProYAB_Calibration__um___IN_SITU_20XTiffVS_Naming_Saving_Copy)

.Run(doc1, doc1)

End With

With Application.WindowCommands.Define(PreProYAB_Calibration__um___IN_SITU_20XTiffVS_Naming_Saving_Copy)

.Run(doc1, window4)

End With

With Application.WindowCommands.Close(PreProYAB_Calibration__um___IN_SITU_20XTiffVS_Naming_Saving_Copy)

.Run(window4)

End With

With Application.DocumentCommands.Activate(PreProYAB_Calibration__um___IN_SITU_20XTiffVS_Naming_Saving_Copy)

.Run(doc5, doc5)

End With

With Application.WindowCommands.QuickSaveSelectedForAnalysis(PreProYAB_Calibration__um___IN_SITU_20XTiffVS_Naming_Saving_Copy)

.Run(varList1)

End With

End Function

**Macro for Segmentation**

Public Function cFOS_Count_YAB_new_measurements_for_INSITU20X() As SimpleScript

cFOS_Count_YAB_new_measurements_for_INSITU20X = New SimpleScript

Dim doc1

With Measure.ThresholdTool.Gadgets.Histogram(cFOS_Count_YAB_new_measurements_for_INSITU20X)

.CheckState = MediaCy.IQL.Application.McCommand.mcCheckState.Checked

.Run()

End With

With Application.DocumentCommands.Active(cFOS_Count_YAB_new_measurements_for_INSITU20X)

.Run(doc1)

End With

With Measure.MeasurementsCommands.Options(cFOS_Count_YAB_new_measurements_for_INSITU20X)

.Segmentation.AutoFindPhase = MediaCy.IQL.Features.mcFindPhase.mcfpManual

.Segmentation.SegmentationType = McMMOptions.mcmmSegmentationType.mcmmstThresholdSegmentation

.Run(doc1)

End With

With Measure.ThresholdToolCommands.Thresholds(cFOS_Count_YAB_new_measurements_for_INSITU20X)

.AllowOverlap = False

.Interpretation = eInterpretation.Mono

.Classes = New System.Collections.Generic.List(Of SegmentationClass)

.Classes.Add(New SegmentationClass("Parent:(none)",System.Drawing.Color.Blue,New Double(){0R,180R}))

.Run(doc1)

End With

With Measure.Measurements.OptionsCommands.Open(cFOS_Count_YAB_new_measurements_for_INSITU20X)

.FileName = "C:\Users\alvarezbagnaryg\Desktop\AOW DAB cFos staining\DAB c-Fos detection whole brain 16um\measurement test.iqo"

.FilterIndex = 1

.Run(doc1)

End With

With Measure.Measurements.HistogramCommands.Options(cFOS_Count_YAB_new_measurements_for_INSITU20X)

.Measurement = New MeasEntry(eMeasures.RgnArea)

.Run(doc1)

End With

With Measure.MeasurementsCommands.ApplyFilters(cFOS_Count_YAB_new_measurements_for_INSITU20X)

.Run(doc1)

End With

With Measure.MeasurementsCommands.CountByROI(cFOS_Count_YAB_new_measurements_for_INSITU20X)

.ClassifyByParent = True

.Run(doc1)

End With

With Measure.Measurements.Gadgets.DataTable(cFOS_Count_YAB_new_measurements_for_INSITU20X)

.CheckState = MediaCy.IQL.Application.McCommand.mcCheckState.Unchecked

.Run()

End With

With Measure.Measurements.Gadgets.DataTable(cFOS_Count_YAB_new_measurements_for_INSITU20X)

.CheckState = MediaCy.IQL.Application.McCommand.mcCheckState.Checked

.Run()

End With

With Measure.MeasurementsCommands.Selection(cFOS_Count_YAB_new_measurements_for_INSITU20X)

.SelectionFlag = McMeasurements.enumMMSelTypes.mcmmsfAddWithReset

.Run(doc1, Nothing)

End With

With Measure.Measurements.TableCommands.Options(cFOS_Count_YAB_new_measurements_for_INSITU20X)

.ShowStatistics = True

.ShowStatisticsOnly = False

.Run(doc1)

End With

End Function
